# Pericytes protect rats and mice from sepsis-induced injuries by maintaining vascular reactivity and barrier function: implication of miRNAs and microvesicles

**DOI:** 10.1186/s40779-023-00442-2

**Published:** 2023-03-13

**Authors:** Zi-Sen Zhang, Yi-Yan Liu, Shuang-Shuang He, Dai-Qin Bao, Hong-Chen Wang, Jie Zhang, Xiao-Yong Peng, Jia-Tao Zang, Yu Zhu, Yue Wu, Qing-Hui Li, Tao Li, Liang-Ming Liu

**Affiliations:** State Key Laboratory of Trauma, Burns and Combined Injury, Department of Shock and Transfusion, Research Institute of Surgery, Daping Hospital, Army Medical University, Chongqing, 400042 China

**Keywords:** Pericyte, Vascular reactivity, Vascular permeability, Cx43, Microvesicle

## Abstract

**Background:**

Vascular hyporeactivity and leakage are key pathophysiologic features that produce multi-organ damage upon sepsis. We hypothesized that pericytes, a group of pluripotent cells that maintain vascular integrity and tension, are protective against sepsis via regulating vascular reactivity and permeability.

**Methods:**

We conducted a series of in vivo experiments using wild-type (WT), platelet-derived growth factor receptor beta (PDGFR-β)-Cre + mT/mG transgenic mice and Tie2-Cre + Cx43^flox/flox^ mice to examine the relative contribution of pericytes in sepsis, either induced by cecal ligation and puncture (CLP) or lipopolysaccharide (LPS) challenge. In a separate set of experiments with Sprague–Dawley (SD) rats, pericytes were depleted using CP-673451, a selective PDGFR-β inhibitor, at a dosage of 40 mg/(kg·d) for 7 consecutive days. Cultured pericytes, vascular endothelial cells (VECs) and vascular smooth muscle cells (VSMCs) were used for mechanistic investigations. The effects of pericytes and pericyte-derived microvesicles (PCMVs) and candidate miRNAs on vascular reactivity and barrier function were also examined.

**Results:**

CLP and LPS induced severe injury/loss of pericytes, vascular hyporeactivity and leakage (*P* < 0.05). Transplantation with exogenous pericytes protected vascular reactivity and barrier function via microvessel colonization (*P* < 0.05). Cx43 knockout in either pericytes or VECs reduced pericyte colonization in microvessels (*P* < 0.05). Additionally, PCMVs transferred miR-145 and miR-132 to VSMCs and VECs, respectively, exerting a protective effect on vascular reactivity and barrier function after sepsis (*P* < 0.05). miR-145 primarily improved the contractile response of VSMCs by activating the sphingosine kinase 2 (Sphk2)/sphingosine-1-phosphate receptor (S1PR)1/phosphorylation of myosin light chain 20 pathway, whereas miR-132 effectively improved the barrier function of VECs by activating the Sphk2/S1PR2/zonula occludens-1 and vascular endothelial-cadherin pathways.

**Conclusions:**

Pericytes are protective against sepsis through regulating vascular reactivity and barrier function. Possible mechanisms include both direct colonization of microvasculature and secretion of PCMVs.

**Supplementary Information:**

The online version contains supplementary material available at 10.1186/s40779-023-00442-2.

## Background

Sepsis and associated multiple organ dysfunction syndrome (MODS) are major causes of mortality in patients with combat injuries [1]. Since vascular dysfunction is a core feature of MODS, many of the treatment strategies focus on vascular dysfunction in patients with sepsis [2, 3]. Despite of decreasing incidence of traumatic sepsis in hospitals over the past two decades, however, the mortality rate in trauma patients remains at ~ 30% [4–6].

Pericytes, a group of perivascular cells, are distributed throughout arterioles, capillaries, and venules [7, 8], and perform vascular-stabilizing and tension-controlling functions [9]. Dysfunction of pericytes contributes to the pathogenesis of a variety of diseases, including diabetic retinopathy, cardiovascular diseases, neurodegenerative diseases and strokes [10, 11]. Pericytes interact with endothelial cells via specific adhesion points, adhesion plaques, gap junctions, and tight junctions [12], and play an important role in endothelial barrier development and integrity maintenance [13]. For instance, within the blood–brain barrier, pericytes contribute to endothelial barrier integrity [14]. Pericyte loss has been shown to aggravate diabetes-induced microvascular dysfunction [15]. A study by Avolio et al. [16] suggested that myocardial pericytes facilitate heart neovascularization during myocardial injury. Additionally, pericyte degeneration leads to changes in cerebrovascular hemodynamics [17]. Collectively, these findings suggest that pericytes play an essential role in maintaining the vascular barrier function and regulating blood flow. However, whether pericytes exert a protective effect on vascular reactivity and barrier function upon sepsis is unknown.

We conducted a series of experiments to investigate the vascular reactivity and barrier functions of pericytes upon sepsis. To this end, we generated platelet-derived growth factor receptor beta (PDGFR-β)-Cre + mT/mG transgenic mice and vascular endothelial cell (VEC)-specific connexin 43 (Cx43) knockout (Tie2-Cre + Cx43^flox/flox^) mice to obtain green fluorescent protein (GFP)-labeled pericytes and to determine the effect of Cx43 on pericyte colonization. Pharmacological depletion of pericytes, cultured mesenteric VECs, and vascular smooth muscle cells (VSMCs) were used to investigate the underlying mechanisms*.*

## Methods

### Animals

Sprague–Dawley (SD) rats were obtained from the Animal Center of Research Institute of Surgery, Army Medical University (Chongqing, China). PDGFR-β-Cre + mT/mG transgenic mice were generated by cross-breeding PDGFR-β-Cre mice (B-CM-004 on the C57BL/6 background, Biocytogen, Beijing, China) with R26^mT/mG^ mice (007576, the Jackson Laboratory, Bar Harbor, ME, USA). PDGFR-β-Cre mice were crossed with mT/mG reporter mice in which Cre-mediated excision resulted in GFP expression. Genotypes were confirmed by polymerase chain reaction (PCR) followed by sequencing (Additional file 1: Fig. S1a, b). Tie2-Cre mice were acquired from Nanjing University (000125, Jiangsu, China). Cx43 in 129S7 Cx43^flox/flox^ mice (008039, the Jackson Laboratory) were conditionally knocked out by Cre/loxP recombinase. Tie2-Cre + Cx43^flox/flox^ mice were generated by cross-breeding Cx43^flox/flox^ mice with Tie2-Cre mice, as previously reported [18, 19]. Genotypes were confirmed by PCR analysis followed by sequencing (Additional file 1: Fig. S1c, d). A total of 896 rats, 144 PDGFR-β-Cre + mT/mG transgenic mice, 104 wild-type (WT) mice (C57BL/6), and 40 Tie2-Cre + Cx43^flox/flox^ mice were used in this study. The study protocol was approved by the Research Council and Animal Care and Use Committee of the Army Medical Center, Army Medical University (AMUWEC20188914). Experiments were conducted in accordance with the Guide for the Care and Use of Laboratory Animals issued by the US National Institutes of Health (NIH Publications, 8th Edition, 2011).

### Pericyte depletion animal model

CP-673451 (S1536, Selleckchem, Houston, TX, USA), a selective PDGFR-β inhibitor, was used to deplete pericytes, as previously described [20]. Briefly, rats received CP-673451 at a dosage of 40 mg/kg per day or vehicle (polyethylene glycol 400) for 7 consecutive days via gastric gavage.

### Preparation of rat and mouse sepsis models

Adult SD rats and mice (8–12 weeks of age) were anesthetized with sodium pentobarbital (45 mg/kg intraperitoneally). Sepsis was induced by cecal ligation and puncture (CLP) or intravenous infusion of lipopolysaccharide (LPS, *Escherichia coli* serotype O111:B4, Sigma), as described previously [21, 22].

For CLP-induced sepsis, laparotomy was performed, and the cecum was exposed, ligated, and punctured 1 cm from the distal end with a triangular needle for rats and three punctures (23-gauge needle) for mice. Feces were allowed to flow into the abdominal cavity. Upon completion of the surgery, the rats and mice were returned to home cages and allowed ad-libitum access to food and water. For LPS-induced sepsis, LPS was injected into the caudal vein at a dosage of 10 mg/kg. Animal subjects with mean arterial pressure at < 70 mmHg or > 30% reduction at 12 h after CLP or LPS administration were used for subsequent experiments. The success rate of the modeling was 89% in this study.

### Isolation and cultivation of VECs and VSMCs

VECs and VSMCs were obtained from the mesenteric veins and arteries of SD rats via enzymatic digestion. Before each experiment, VECs and VSMCs (3–5 passages) were serum-starved for 24 h.

### Isolation, cultivation, and identification of pericytes

Pericytes were isolated and cultured as previously described [23]. Morphological characterization was performed using phase contrast microscopy, and immunofluorescence characterization was performed using confocal laser scanning microscopy (CLSM; SP5II, Leica Microsystems, Wetzlar, Germany). The cells were verified with primary antibodies against PDGFR-β (ab32570, Abcam, Cambridge, UK), nerve/glial antigen 2 (NG-2; ab5320, Merck Millipore, Burlington, MA, USA), CD146 (ab75769, Abcam), α-smooth muscle actin (α-SMA; ab7817, Abcam), and platelet endothelial cell adhesion molecule (CD31; ab24590, Abcam).

For flow cytometry, cells were labeled with directly conjugated antibodies, including NG-2-PE, CD146-PE, PDGFR-β-PE, CD31-PE and IgG-PE (all from BD Biosciences, Franklin Lakes, NJ, USA). Samples were analyzed using the high-sensitivity imaging flow cytometer Amnis ImageStream MK II (IS^X^).

### Pericyte transplantation

Animal subjects in the control group received conventional treatment of sepsis animals, including fluid resuscitation [lactated Ringer’s solution (LR) 35 ml/kg], vasopressor (dopamine 1.75 mg/kg), and antibiotics (cefuroxime sodium, 100 mg/kg) at 12 h after CLP [6]. Pericytes were primed for 24 h with or without polyinosine–polycytidylic acid [Poly(I:C), 20 μg/ml; P1530, Sigma], and infused at a dosage of 1 × 10^6^ pericytes slowly in 200-μl saline via the femoral vein at 12 h after sepsis. To visualize pericyte colonization, pericytes were transfected with Cx43 shRNA adenovirus (PC^Cx43−down^) or control adenovirus expressing GFP (PC^vehicle^, Genechem Technologies, Shanghai, China).

### Vascular reactivity

The abdomen was opened via a midline incision. The ileocecal portion of the mesentery was gently exteriorized and mounted on a transparent plastic stage under moist condition at 37 °C. Single unbranched arterioles without obvious bends, with diameters ranging from 30 to 50 μm, and lengths of approximately 200 µm were used to determine responses to norepinephrine (NE) at 10^–7^ to 10^–4^ mol/L and acetylcholine (Ach) at 10^–3^ mol/L [24]. Changes in arteriole diameter were recorded with a video camera (OLYMPUS, DP21, Tokyo, Japan) and analyzed using Image-Pro Plus 5.0 software (Media Cybernetics Inc., Rockville, MD, USA). Contraction was calculated as (D_Baseline_ – D_NE_)/D_Baseline_ × 100%; dilation was calculated as (D_Ach10_^−3^ – D_NE10_^−4^)/(D_Baseline_ – D_NE10_^−4^) × 100% (Additional file 2: Video S1).

### Vascular permeability

Fluorescein isothiocyanate (FITC)–bovine serum albumin (BSA) was used to evaluate albumin leakage across the mesenteric venular wall using inverted intravital microscopy (C11440, Hamamatsu, Shizuoka, Japan). Briefly, under anesthesia, the rat abdomen was opened via a midline incision. The ileocecal portion of the mesentery (10 to 15 cm from the caudal mesentery) was exteriorized and mounted on a transparent plastic stage under moist condition at 37 °C. Fluorescence intensity in the venules (IV) and perivenular interstitium (IP) was recorded at 0, 1, 3, and 6 min after an intravenous injection of FITC–BSA (50 mg/kg) using Image-Pro Plus 5.0 software. FITC–BSA leakage was estimated by dividing IP by IV, and the ratio of FITC–BSA leakage at a given time point to that of the baseline was designated as the ratio of FITC–BSA leakage at that point. For the mesenteric microvessel networks measurement, 50 mg/kg FITC–BSA was injected intravenously and allowed to circulate for 5 min; the fluorescence intensity of FITC–BSA was recorded and the FITC–BSA^+^ area per vessel was quantified.

### Immunohistochemistry of rat mesenteric microvessels

Mesenteric tissues were harvested and fixed in 4% paraformaldehyde at 4 °C overnight. Samples were extensively washed with phosphate-buffered saline (PBS) and incubated for 30 min at 37 °C with 0.1% Triton X-100 in PBS, and blocked with 5% BSA prior to incubation with antibodies against PDGFR-β, NG-2, vascular endothelial (VE)-cadherin (555289; BD Biosciences), zonula occludens-1 (ZO-1; 33–9100, Invitrogen, Waltham, MA, USA), and CD31.

### Electron microscopy

The mesenteric venules were fixed in 3% glutaraldehyde in 0.1 mol/L PBS for 20 min, cut into blocks smaller than 1 mm^3^ and then post-fixed by immersion in the same fixative for 1 h at room temperature and then overnight at 4 °C. Samples were incubated in 1% osmium tetroxide in 0.1 mol/L PBS for 2 h at 4 °C, dehydrated, and then embedded in Epon 812. Ultrathin sections were stained with uranyl acetate and lead citrate for observation using transmission electron microscopy (TEM; JEM 1400, JEOL Ltd., Tokyo, Japan).

### Quantification of microvesicles

Microvesicles were isolated from culture supernatant with successive centrifugations [25]. Briefly, the medium was centrifuged at 1500 *g* for 5 min. The supernatant was centrifuged at 16,000 *g* for 1 h. The pellet was resuspended in 1 ml PBS and centrifuged at 16,000 *g* for 45 min. The process was repeated twice, and the final pellet was suspended in PBS and stored at -80 °C until use. Negative staining and ultrathin sections were used for TEM analysis.

For negative staining, microvesicle preparations were measured as previously described [26]. Briefly, the microvesicles preparations were fixed in 2.5% glutaraldehyde in PBS at 4 °C for 24 h. After rinsing twice with 0.1 mol/L PBS, samples were post-fixed in 1% OsO_4_ at room temperature for 70 min. After rinsing thrice with 0.1 mol/L PBS, samples were dehydrated using a series of graded ethanol. Finally, the samples were embedded in Epon 812 and 100 nm sections were prepared on grids. The microvesicles were analyzed using TEM (JEM 1400, JEOL Ltd.).

For scanning electron microscopy (SEM) analysis, the microvesicles preparations were fixed with 2.5% glutaraldehyde overnight, washed 2–3 times with 0.1 mol/L PBS, dehydrated with a series of graded ethanol (5 min in 30%, 5 min in 50%, 10 min in 70%, 10 min in 90%, and twice for 10 min in absolute ethanol), and dried with CO_2_ using the critical point method with a dryer. Dried samples were covered with a 10-nm gold layer and scanned using a Zeiss Crossbeam 340 electron microscope.

For dynamic light scattering (DLS) analysis, the microvesicles preparations were suspended in 1 ml of PBS and then loaded into a cuvette for DLS analysis using a Zetasizer Nano ZS (Malvern Instruments, Ltd., Worcestershire, UK) at room temperature with a 633-nm He–Ne laser automatic attenuator. Each sample was measured at least three times.

For flow cytometry analysis, 10 μl of 0.2 μm, 0.5 μm, or 0.8 μm standard microbeads were added to 100 µl of PBS, respectively. Microvesicles preparations were suspended in 100 μl of PBS; 10 μl of 10 × Annexin V-binding buffer (10 mmol/L HEPES, pH 7.4, 140 mmol/L NaCl, 2.5 mmol/L CaCl_2_) and 5 μl of APC-Annexin V were added to microvesicle preparations. Annexin V was used to detect total microvesicles. After incubation for 25 min in the dark at room temperature, the samples were analyzed using the high-sensitivity imaging flow cytometer Amnis ImageStream MK II (IS^X^).

### Endocytosis of PCMVs by VSMCs and VECs

PKH-26 (MiNi26-1KT, Sigma), a red fluorescent dye that binds to the lipid bilayer, was used to label PCMVs [27]. Briefly, PCMVs were stained with PKH-26 dye in 0.4 ml of diluent C fluid for 5 min at room temperature. An equal volume of PCMV-depleted serum was used to stop the labeling reaction. Next, 5 ml of serum-free medium was added, and unbound PKH-26 was removed using centrifugation at 20,000 *g* for 40 min. VECs and VSMCs were subsequently incubated with 5 µl of labeled PCMVs for 0, 4, and 12 h in glass-bottom cell culture dishes at 37 °C and then washed with PBS. The uptake of labeled PCMVs by VECs and VSMCs was determined using CLSM.

### Co-culture of VSMCs and VECs with PCMVs

Transendothelial electrical resistance (TEER) and penetration rate in VECs were measured as previously described [28]. Briefly, VECs were seeded on inserts (100,000 cells per well) in a 6-well culture plate (0.4-μm pore size, 3450, Corning Inc., Corning, NY, USA). After 12 h exposure to LPS (2 μg/ml), the VECs in the PCMV group were incubated with PCMVs (2 × 10^6^/ml) for 24 h. TEER was determined using a voltohmmeter (World Precision Instruments Inc., Sarasota, FL, USA) at 30 min interval. For penetration rate, FITC–BSA (10 μg/ml; A9771, Sigma) was added to the inserts, and 200 μl of the supernatant was collected every 10 min with 200 μl fresh basal medium. The penetration rate was calculated based on the total supernatant fluorescence OD/FITC–BSA stain fluorescence OD.

### Contraction in cultured VSMCs

Contraction of cultured VSMCs was determined as previously described [29]. Briefly, VSMCs (3–5 passage) were plated on collagen-coated polyethylene terephthalate cell culture inserts (3 μm pore, 3452, Corning) in 24-well culture plates. The lower compartment of the Transwell was filled with 600 μl of medium and cultured for 48 h. After 12 h LPS exposure, the VSMCs in the PCMV group were incubated with PCMVs (2 × 10^6^) for 24 h. The contractile response of VSMC to NE was determined by measuring the infiltration ratio of FITC–BSA.

### 3D cell culture and assessment of contact area

For 3D imaging, GFP-VECs/VSMCs (control adenovirus) were used to distinguish VECs/VSMCs exhibiting GFP, whereas pericytes were labeled with mCherry to distinguish pericytes exhibiting red fluorescence. Briefly, GFP-VECs/VSMCs were cultured with mCherry-pericytes (control adenovirus) or PC^Cx43−down^ (Cx43 shRNA adenovirus) for 24 h. GFP-VECs/VSMCs and mCherry-pericytes were observed within a 3D volume viewer using CLSM.

### Quantification of miRNAs

miRNAs were extracted from the PCMVs using the miRCute™ RNA Isolation Kit (RP5301, BioTeke, Beijing, China). Reverse transcription (RT)-PCR was performed using the All-in-One™ miRNA qPCR Detection Kit (GeneCopoeia, Rockville, MD, USA) on a C1000™ Thermal Cycler Real-Time PCR system from Applied Biosystems (Bio-Rad, Hercules, CA, USA). The induction was calculated using the Ct method as follows: ΔΔCt = (Ct target miRNA − Ct U6), and the final values were determined using 2^−ΔΔCt^.

### miRNA transfection

Cells were transfected with a miR-145/132 inhibitor, a miR-145/132 mimic or a control miRNA (EF013, GeneCopoeia) [30]. miR-145/132-downregulated PCMV [miR-145/132(-)PCMV], miR-145/132-upregulated PCMV [miR-145/132(+)PCMV], or vehicle-PCMV were isolated from the supernatant of miR-145/132(-)-pericyte and miR-145/132(+)-pericyte or vehicle-pericyte.

### Western blotting analysis

Western blotting analysis was performed using antibodies against the following: PDGFR-β (1:1000, ab32570, Abcam), NG-2 (1:1000, ab129051, Abcam), VE-cadherin (1:1000, ab231227, Abcam), ZO-1 (1:1000, ab190085, Abcam), p-MLC_20_ (1:2000, M6068, Sigma), MLC_20_ (1:2000, 3672, Cell Signaling Technology, Danvers, MA, USA), Sphk2 (1:2000, PA5-99,720, Thermo Fisher Scientific, Waltham, MA, USA), S1PR1 (1:2000, ab11424, Abcam), S1PR2 (1:2000, PA5-72,868, Thermo Fisher Scientific), and β-actin (1:7000, A5441, Sigma). Bands were detected with fluorescent secondary antibodies and quantified using the Odyssey CLx Infrared Imaging System (LI-COR, Lincoln, NE, USA).

### Statistical analysis

The results are expressed as mean ± standard deviation for the indicated number of experiments. Student’s *t*-test was used for statistical analysis between two groups, and one-way analysis of variance (ANOVA) for comparisons involving three or more groups, followed by Tukey’s post hoc test for pairwise comparisons. Survival data were analyzed using the log-rank test (Kaplan–Meier curves). All statistical analyses were conducted using the SPSS software (version 11.0). Statistical significance was set at *P* < 0.05 (2-sided for all analyses).

## Results

### Pericyte loss and destruction contribute to vascular hyporeactivity and vascular leakage following sepsis

**Fig. 1:**
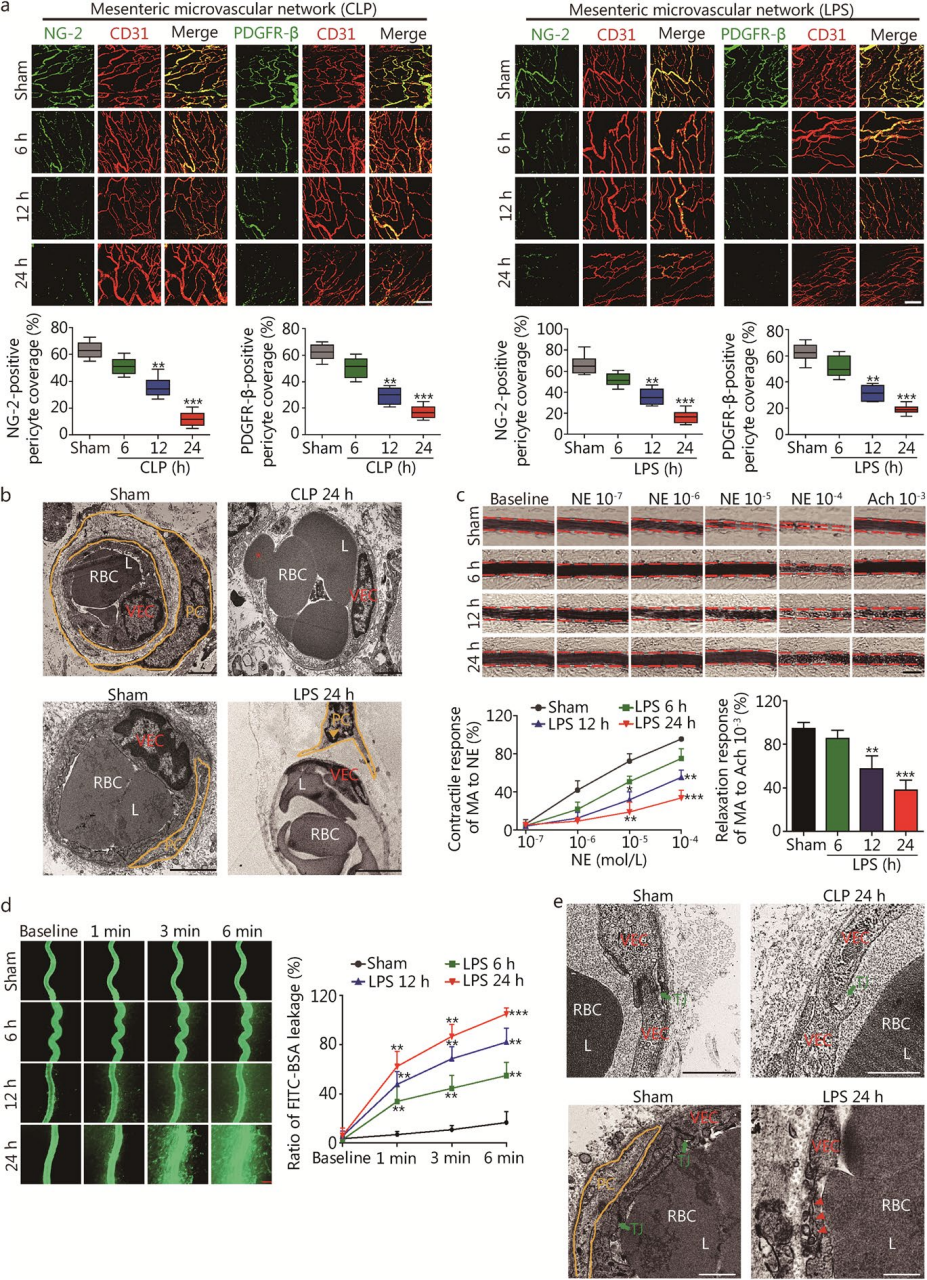
Sepsis induces pericyte loss, vascular hyporeactivity and leakage in rats. **a** Mesenteric microvascular networks from CLP and LPS (10 mg/kg)-induced sepsis at 6, 12 and 24 h were stained for NG-2 (pericyte marker; green), PDGFR-β (pericyte marker; green), and CD31 (VEC marker; red). Pericyte coverage rate of endothelium was quantified by analyzing percentage of CD31^+^ capillaries opposed to NG-2^+^ and PDGFR-β^+^ PCs (*n* = 8 rats). Scale bars: 100 μm. **b** TEM observation of ultrastructural changes of pericyte in mesenteric venules at 24 h after CLP and LPS administration (yellow arrowheads indicate pericyte loss and swelling, *indicate erythrocyte diapedesis). Scale bars: 2 μm.** c** Changes in vascular response of mesenteric arterioles to NE and Ach in vivo (*n* = 8 rats). **d** Vascular leakage of mesenteric venules measured by the appearance of intravenously injected FITC–BSA and quantitation of FITC–BSA^+^ vessel (*n* = 8 rats). Scale bars: 50 μm. **e** Representative TEM images of tight junctions in mesenteric venules after CLP and LPS administration at 24 h (green arrow indicate the tight junction, red arrowheads indicate the endothelial fragments and disrupted VEC junctions). Scale bars: 1 μm. PC pericyte, CLP cecal ligation and puncture, LPS lipopolysaccharides, NG-2 nerve/glial antigen 2, PDGFR-β platelet-derived growth factor receptor beta, VEC vascular endothelial cell, RBC red blood cell, L lumen, NE norepinephrine, Ach acetylcholine, MA mesenteric arteriole, TJ tight junction, TEM transmission electron microscopy. Data shown as mean ± SD. ^*^P < 0.05, ^**^P < 0.01, ^***^P < 0.001 vs. Sham (one-way ANOVA)

Pericytes were severely damaged following sepsis in CLP- and LPS-treated rats. Immunofluorescence staining showed significantly decreased expression of pericyte markers (NG-2 and PDGFR-β) in the mesenteric microvascular networks and mesenteric venules at 12 and 24 h after either CLP or LPS in rats (*P* < 0.01, Fig. 1a, Additional file 1: Fig. S2a). Decreased expression of NG-2 and PDGFR-β was also evident in Western blotting analysis (*P* < 0.01, Additional file 1: Fig. S2b). Electron microscopy showed that pericytes were tightly ensheathed within the endothelium in the sham control group, but severe desquamation, swelling and destruction in the mesenteric venules and retina (Fig. 1b, Additional file 1: Fig. S2c), and diapedesis in the mesenteric venules at 24 h in CLP rats (Fig. 1b).

Responses of mesenteric arterioles to both NE and Ach were significantly decreased at 12 and 24 h (*P* < 0.05, Fig. 1c). The expression of p-MLC_20_, a regulatory protein of vascular smooth muscle in the superior mesenteric artery (SMA), was significantly decreased (*P* < 0.05, Additional file 1: Fig. S2d). Permeability of the mesenteric venules and microvascular networks started to increase at 1 min after LPS exposure, and became more pronounced at 6 min (*P* < 0.01, Fig. 1d, Additional file 1: Fig. S2e). Electron microscopy showed disrupted continuity and integrity in the VECs of mesenteric venules (Fig. 1e) and severe swelling in the retina at 24 h after sepsis (Additional file 1: Fig. S2c). Immunohistochemical staining of ZO-1 and VE-cadherin revealed defective tight and adhesion junctions at 24 h after sepsis (Additional file 1: Fig. S2f). Western blotting analysis showed decreased expression of ZO-1 and VE-cadherin in superior mesenteric veins (SMVs) at 12 and 24 h in CLP and LPS rats (*P* < 0.01, Additional file 1: Fig. S2g).

Similar to SD rats, fluorescent-labeled pericytes in PDGFR-β-Cre + mT/mG transgenic mice also exhibited significant destruction and desquamation after sepsis (Additional file 1: Fig. S3a). Electron microscopy revealed severe damage to pericytes in the mesenteric venules and retina of mice after sepsis (Additional file 1: Figs. S3b). Changes in vascular reactivity and permeability were consistent with those in septic rats (Additional file 1: Fig. S3c-f).

### Pericyte transplantation protects vascular reactivity and barrier function in septic rats

Next, we examined the potential effects of transplantation of cultivated pericytes (Additional file 1: Fig. S4a-c) and function-enhanced [Poly(I:C) pre-treatment] pericytes [Poly(I:C)PC] in septic rats.

#### Effect of pericyte transplantation

**Fig. 2:**
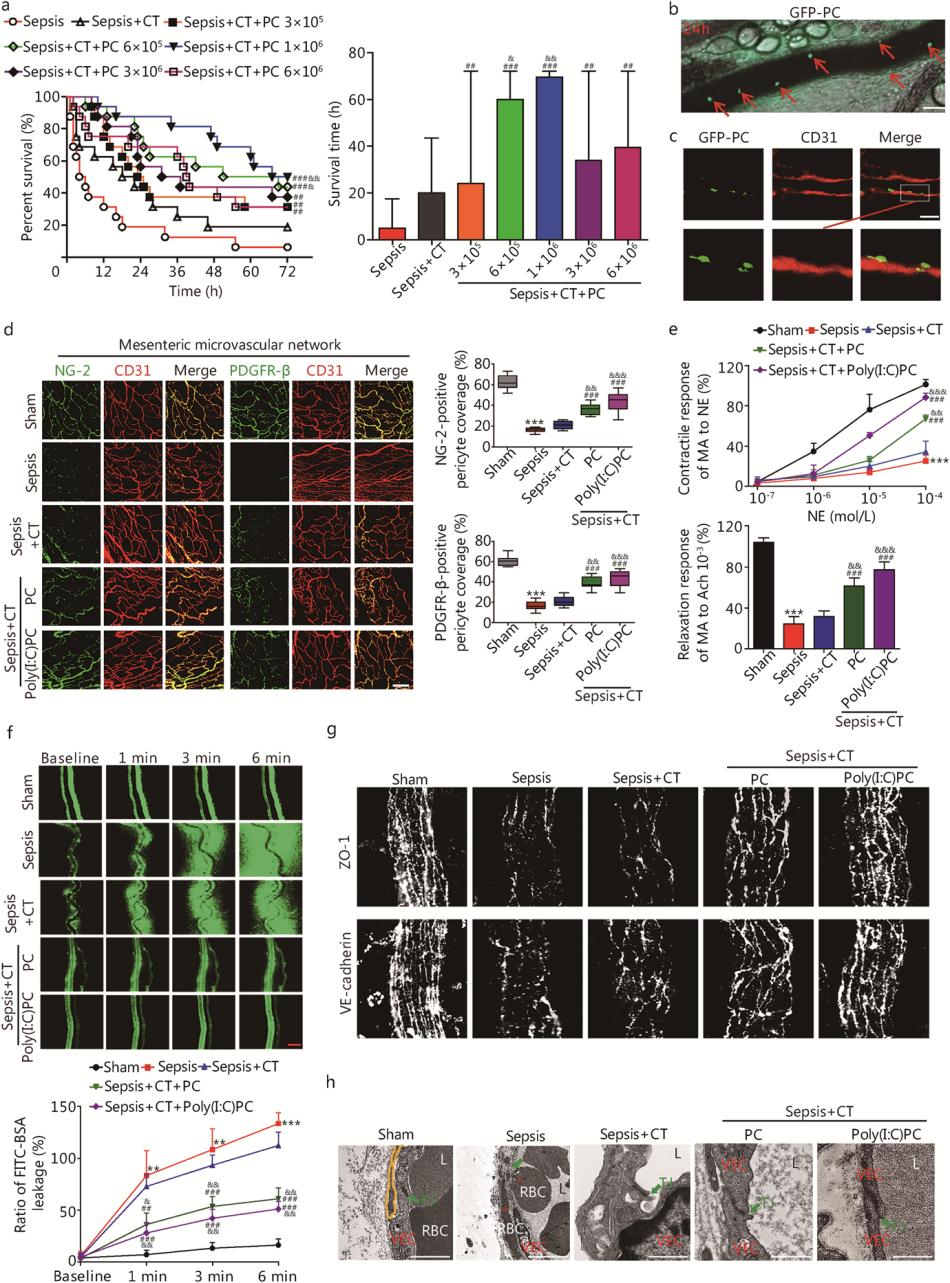
The transplanted pericytes improve the vascular hyporeactivity and leakage after sepsis. **a** Effects of transplanting different amount of exogenous pericytes on animal survival (*n* = 16 rats). Intravital microscopy (**b**, red arrows indicate GFP-PC) and immunofluorescence (**c**) by CLSM were used to monitor the GFP-PC location on mesenteric venules at 24 h after transplantation of exogenous pericytes (10^6^). Scale bars: 50 μm. **d** Mesenteric microvascular networks were stained for NG-2, PDGFR-β, and CD31 at 24 h after resuscitation (*n* = 8 rats). Scale bars: 100 μm.** e** Changes in vascular response of mesenteric arterioles to NE and Ach in vivo after sepsis in rats (*n* = 8). **f** Vascular leakage of mesenteric venules measured by the appearance of intravenously injected FITC–BSA and quantitation of FITC–BSA^+^ vessel (*n* = 8 rats). Scale bars: 50 μm. **g** Immunohistochemistry for ZO-1 and VE-cadherin in mesenteric venules. Scale bars: 20 μm. **h** Representative TEM images of tight junctions in mesenteric venules (green arrows indicate the tight junction, *indicate the erythrocyte diapedesis). Scale bars: 1 μm. NG-2 nerve/glial antigen 2, PDGFR-β platelet-derived growth factor receptor beta, CT conventional treatment, CLSM confocal laser scanning microscopy, PC pericyte, Poly(I:C)PC polyinosine-polycytidylic acid pre-treatment pericyte, NE norepinephrine, Ach acetylcholine, MA mesenteric arteriole, ZO-1 zonula occludens-1, VE-cadherin vascular endothelial cadherin, VEC vascular endothelial cell, RBC red blood cell, TJ tight junction, L lumen, TEM transmission electron microscopy. Data shown as mean ± SD. ^**^P < 0.01, ^***^P < 0.001 vs. Sham; ^##^*P* < 0.01, ^###^*P* < 0.001 vs. Sepsis; ^&&^*P* < 0.01, ^&&&^*P* < 0.001 vs. Sepsis + CT (one-way ANOVA)

Pericyte transplantation (1 × 10^6^) increased the 72-h survival rate in septic rats [50.0% (8/16) vs. 18.8% (3/16) in the conventional treatment group and 6.3% (1/16) in the sepsis group, *P* < 0.01, Fig. 2a]. GFP-labeled pericytes (Additional file 1: Fig. S4d) were found to colonize in the mesenteric venules at 6, 12, 24 and 36 h after pericyte transplantation (10^6^), with the highest rate of colonization at 24 h (Fig. 2b, Additional file 1: Fig. S4e). Notably, the transplanted pericytes were embedded within the vascular endothelium of mesenteric venules (Fig. 2c).

Pericyte transplantation improved vascular reactivity and barrier function in septic rats (*P* < 0.05, Additional file 1: Fig. S4f–i); observed effects were noticed as early as 6 h, and reached plateau at 24 h. Both NG-2 and PDGFR-β were upregulated in the pericyte and Poly(I:C)PC groups (*P* < 0.01; Fig. 2d, Additional file 1: Fig. S5a, b). The conventional treatment group exhibited only marginal improvements in vascular function. Protective effects were significant in both the pericyte and Poly(I:C)PC groups (*P* < 0.01 vs. both the sepsis and conventional treatment groups, Fig. 2e-f, Additional file 1: Fig. S5c, d).

Pericyte transplantation improved the integrity of tight and adhesion junctions (Fig. 2g) as well as the ultrastructure of tight junctions (Fig. 2h, Additional file 1: Fig. S5e). The expression levels of p-MLC_20_ in SMA, ZO-1 and VE-cadherin in SMV were significantly increased (*P* < 0.01, Additional file 1: Fig. S5f, g).

#### Pericyte colonization is associated with Cx43

**Fig. 3:**
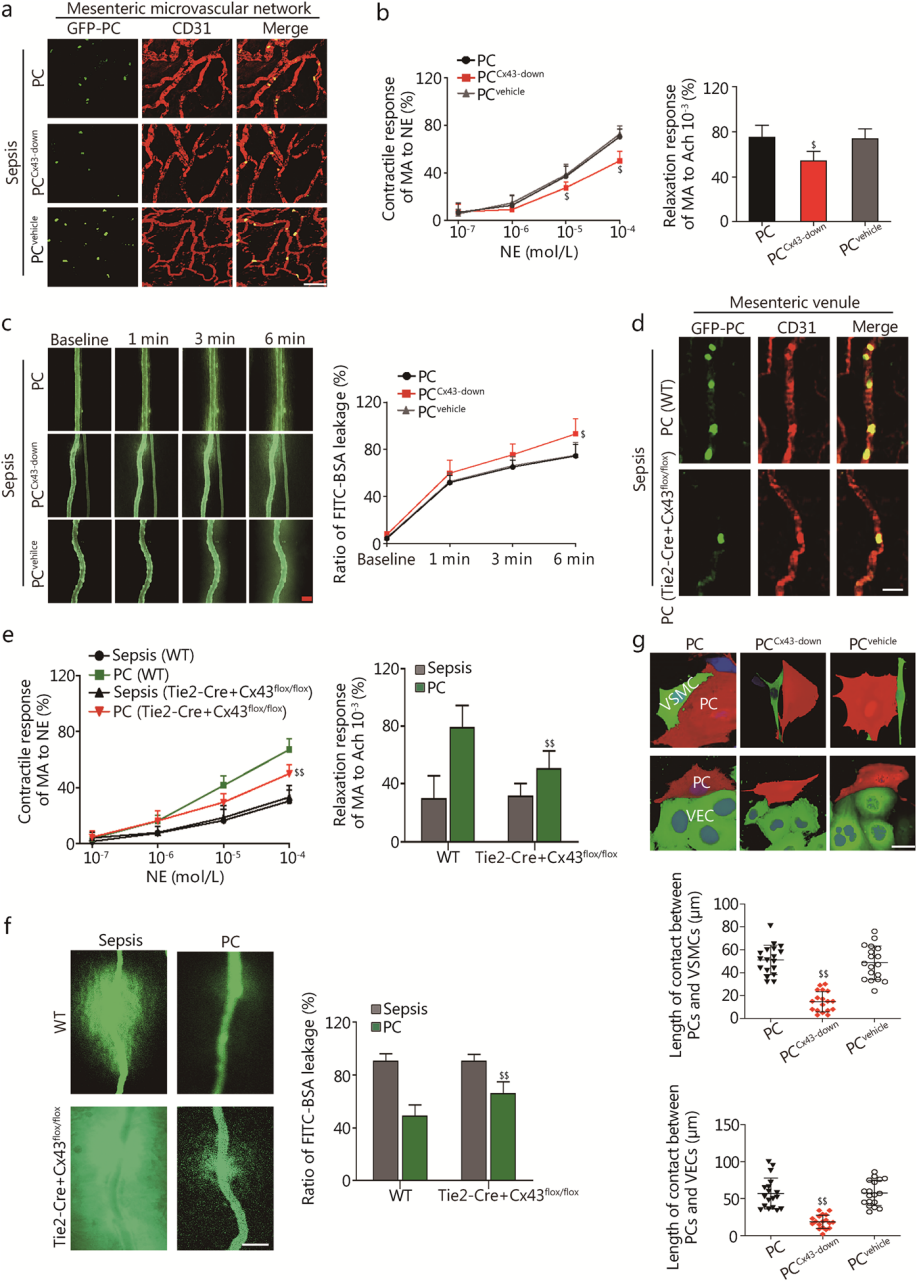
Transplanted pericytes regulate vascular reactivity and permeability via Cx43 after sepsis. **a** Immunofluorescence by CLSM was used to monitor the PC^Cx43−down^ colonization on mesenteric microvascular networks in septic rats. Scale bars: 100 μm.** b** Changes in vascular response of mesenteric arterioles to NE and Ach in vivo after PC^Cx43−down^ transplantation (*n* = 8 rats).** c** Vascular leakage of mesenteric venules measured after PC^Cx43−down^ transplantation (*n* = 8 rats). Scale bars: 50 μm. **d** Immunofluorescence by CLSM was used to monitor the GFP-PC location on mesenteric venules at 24 h after sepsis in Tie2-Cre + Cx43^flox/flox^ mice. Scale bars: 20 μm. **e** Changes in vascular response of mesenteric arterioles to NE and Ach in vivo after sepsis in Tie2-Cre + Cx43^flox/flox^ mice (*n* = 8). **f** Vascular leakage of mesenteric venules measured after sepsis in Tie2-Cre + Cx43^flox/flox^ mice (*n* = 8). Scale bars: 20 μm. **g** 3D projection images of contact area on 24 h in the pericytes-VSMCs/VECs culture at a 1:9 pericyte:VSMCs/VECs ratio (PC group: pericyte with no-treatment; PC^Cx43−down^ group: infection of pericytes with shRNA adenovirus targeting Cx43; PC^vehicle^ group: infection of pericytes with control adenovirus). Pericytes, VSMCs/VECs and nuclei are shown in red, green and blue, respectively. Scale bars: 20 μm. CLSM confocal laser scanning microscopy, PC pericyte, NE norepinephrine, Ach acetylcholine, MA mesenteric arteriole, VECs vascular endothelial cells, VSMCs vascular smooth muscle cells. Data shown as mean ± SD. ^$^*P* < 0.05, ^$$^*P* < 0.01 vs. PC or PC (WT) (one-way ANOVA)

To examine the potential role of Cx43 in the colonization of microvessels by transplanted pericytes, we conducted a series of experiments in Cx43 shRNA adenovirus-infected pericytes (PC^Cx43−down^) and VEC Cx43-knockout mice (Tie2-Cre + Cx43^flox/flox^ mice). The results showed significantly lower number of colonizing PC^Cx43−down^ in the mesenteric microvascular networks than in the pericyte group (Fig. 3a). PC^Cx43−down^ transplantation showed decreased protective effect on vascular reactivity and barrier function in septic rats, compared to pericyte group (*P* < 0.05, Fig. 3b, c; Additional file 1: Fig. S6a-c).

In comparison to the WT mouse control, the extent of transplanted pericyte colonization as well as the associated protective effects on vascular reactivity and vascular barrier function were significantly reduced in Tie2-Cre + Cx43^flox/flox^ mice (*P* < 0.05, Fig. 3d-f, Additional file 1: Fig. S6d).

In the 3D VSMCs/pericytes or VECs/pericytes co-culture, pericytes formed direct connection with VSMCs or VECs via Cx43 (Additional file 1: Fig. S6e). Knockdown of Cx43 in pericytes reduced the contact area (*P* < 0.05, Fig. 3g; Additional files 3–8: Videos S2-S7) and number of cells in contact with each other (*P* < 0.01, Additional file 1: Fig. S6f).

### Pericyte-depleted rats are recapitulated by CP-673451

Repeated CP-673451 treatment for 7 consecutive days, a PDGFR inhibitor, reduced the amount of vascular pericytes within the rat mesentery and decreased the expression of pericyte markers (NG-2 and PDGFR-β) in mesenteric microvascular networks (Additional file 1: Fig. S7a, b). Electron microscopy revealed reduced pericyte in mesenteric venules (Additional file 1: Fig. S7c). In contrast, the heart, lung, liver, and kidney functions were not altered. CP-673451 treatment for 14 consecutive days also damaged heart and kidney functions in SD rats in addition to depletion of pericytes in the mesenteric microvessels (Additional file 1: Fig. S7d, e). Accordingly, the 7-day-CP-673451 treatment regimen was used in subsequent experiments.

### Pericyte depletion aggravates sepsis-induced vascular hyporeactivity and vascular leakage, which is rescued by pericyte transplantation

Repeated CP-673451 treatment (40 mg/kg) for 7 d resulted in pericyte loss within the mesenteric microvascular networks. Both NG-2 and PDGFR-β were down-regulated (*P* < 0.001, Additional file 1: Fig. S8). Vascular reactivity and vascular barrier function were markedly impaired (*P* < 0.001, Additional file 1: Figs. S9 and S10). Pericyte transplantation restored pericyte coverage, vascular reactivity, and barrier function to normal levels (Additional file 1: Figs. S8-S10).

In septic rats, pericyte depletion aggravated sepsis-induced pericyte loss (*P* < 0.01, Additional file 1: Fig. S8), vascular hyporeactivity and permeability damage (*P* < 0.01, Additional file 1: Figs. S9 and S10). Electron microscopy revealed fragmentation of the endothelial cell membrane, disruption of tight junctions, and erythrocyte diapedesis within the mesenteric venules (Additional file 1: Fig. S10d). Pericyte transplantation rescued the poor pericyte coverage (*P* < 0.01, Additional file 1: Fig. S8), damaged vascular reactivity (*P* < 0.01, Additional file 1: Fig. S9) and barrier function (*P* < 0.01, Additional file 1: Fig. S10). The rescue effects were more pronounced with Poly(I:C)-treated pericytes (*P* < 0.01, Additional file 1: Figs. S8-S10).

### Pericytes may transfer miRNAs to VSMCs and VECs via microvesicles to regulate and protect vascular reactivity and barrier function after sepsis

#### Role of PCMVs in cultured VSMCs and VECs in vitro

**Fig. 4:**
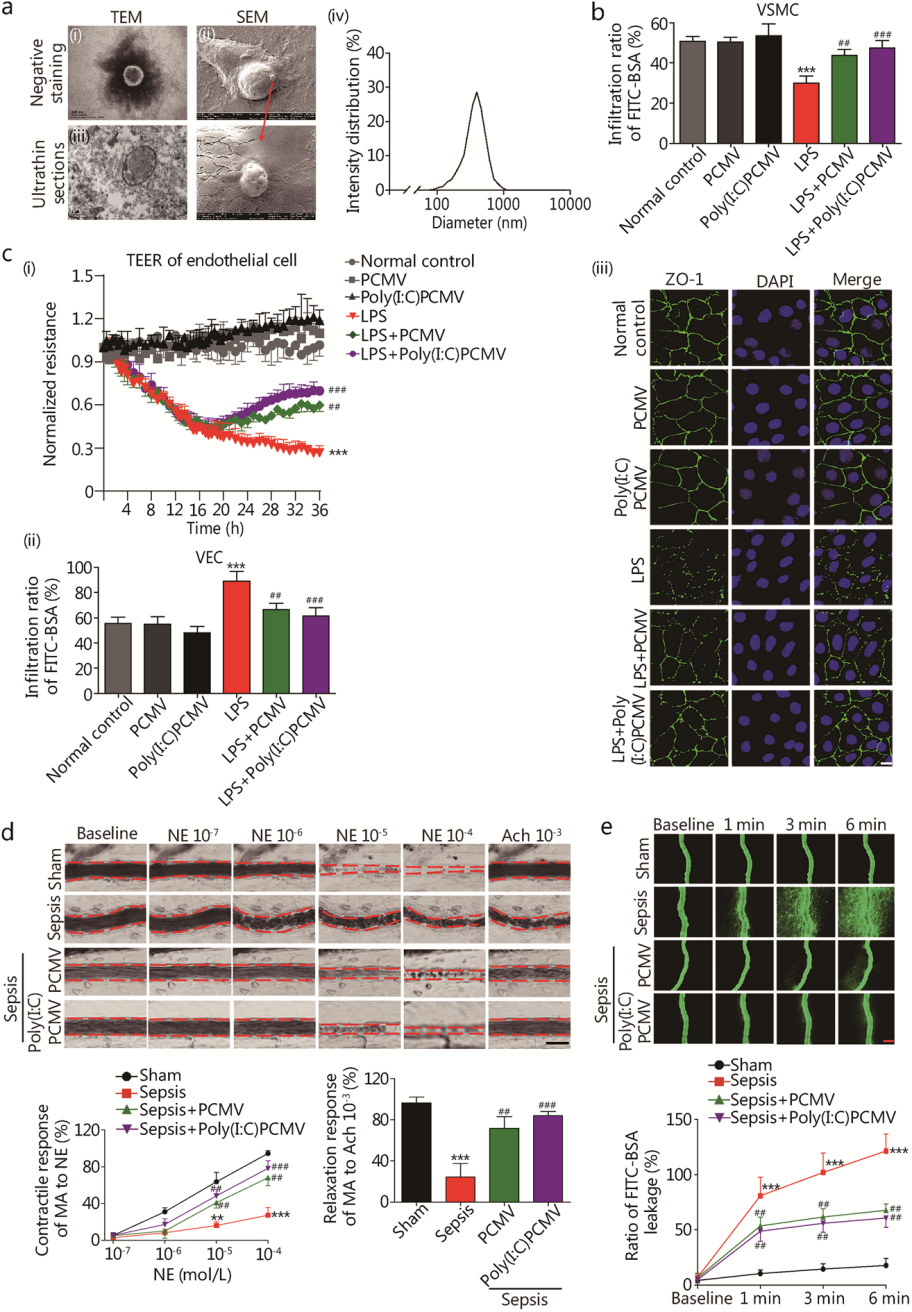
PCMVs regulate the contractile response of VSMCs and barrier function of VECs after sepsis. **a** Identification of PCMV. (**i-ii**) Representative TEM micrographs of microvesicle isolated from pericyte; (**iii**) Representative SEM micrographs of microvesicle observed from pericytes; (**iv**) PCMV diameter measured by DLS analysis. **b** Role of PCMVs and Poly(I:C)PCMVs (2 × 10^6^ microvesicles/ml) on the contractile response of rat VSMC to NE at 12 h after LPS (2 μg/ml) stimulation (*n* = 8 cells). **c** Role of PCMVs and Poly(I:C)PCMVs on the barrier function of rat VECs after LPS administration. **i** PCMVs and Poly(I:C)PCMVs were added into rat VECs, and TEER of each group was measured (*n* = 3 cells); **ii** PCMVs and Poly(I:C)PCMVs were added into VECs, and FITC–BSA penetration of each group was measured (*n* = 8 cells); **iii** VECs treated with PCMV were analyzed by immunofluorescence for ZO-1. Scale bars: 20 μm. **d** Changes in vascular response of mesenteric arterioles to NE and Ach in vivo after PCMV transplantation (*n* = 8 rats). Scale bars: 50 μm.** e** Vascular leakage of mesenteric venules measured after PCMV transplantation (*n* = 8 rats). Scale bars: 50 μm. PC pericyte, PCMV pericyte-derived microvesicle, TEM transmission electron microscopy, SEM scanning electron microscopy, VECs vascular endothelial cells, VSMCs vascular smooth muscle cells, LPS lipopolysaccharides, TEER transendothelial electrical resistance, ZO-1 zonula occludens-1, NE norepinephrine, Ach acetylcholine, MA mesenteric arteriole. Data shown as mean ± SD. ^**^P < 0.01, ^***^P < 0.001 vs. Normal control or Sham; ^##^*P* < 0.01, ^###^*P* < 0.001 vs. LPS or Sepsis (one-way ANOVA)

Although pericyte colonization was highest 24 h after transplantation, protective effects on vascular function were observed within the first 6 h. Considering our previous study showing that pericytes can release microvesicle to deliver connective tissue growth factors to VECs and promote their proliferation [31], we speculated that the early effects caused by pericytes are associated with PCMV release. Indeed, pericytes secreted PCMVs with characteristics similar to other cell-derived microvesicles (for example, 100–1000 nm in diameter) (Fig. 4a, Additional file 1: Fig. S11a). Poly(I:C)-treated pericytes released even more PCMVs. PKH-26-labeling suggested that PCMVs entered VECs and VSMCs in a time-dependent manner (Additional file 1: Fig. S11b). Additionally, PCMV (2 × 10^6^ microvesicles/ml, 12 h after LPS stimulation) incubation alleviated LPS-induced damage to VSMC contractile function and VEC barrier function. PCMVs also improved the contractile response of VSMCs to NE and the expression of p-MLC_20_ (*P* < 0.05, Fig. 4b, Additional file 1: Fig. S11c), while improving the integrity of VECs and expression of ZO-1 and VE-cadherin (*P* < 0.05, Fig. 4c, Additional file 1: Fig. S11c). The protective effects of Poly(I:C)-induced PCMVs on VSMC contractile function and VEC barrier function were even stronger.

#### Role of PCMVs in septic rats in vivo

Similar to the in vitro findings, PCMV infusion (2 × 10^7^ microvesicles per rat, at 12 h after sepsis [31]) significantly improved the vascular reactivity and barrier function in septic rats at 24 h (*P* < 0.05, Fig. 4d, e; Additional file 1: Fig. S11d-g). The vasoconstriction and dilation responses of the mesenteric arteriole to NE were significantly increased. Vascular permeability and integrity of ZO-1 and VE-cadherin were also improved.

#### Role of miRNAs transferred by PCMVs to VSMCs and VECs

**Fig. 5:**
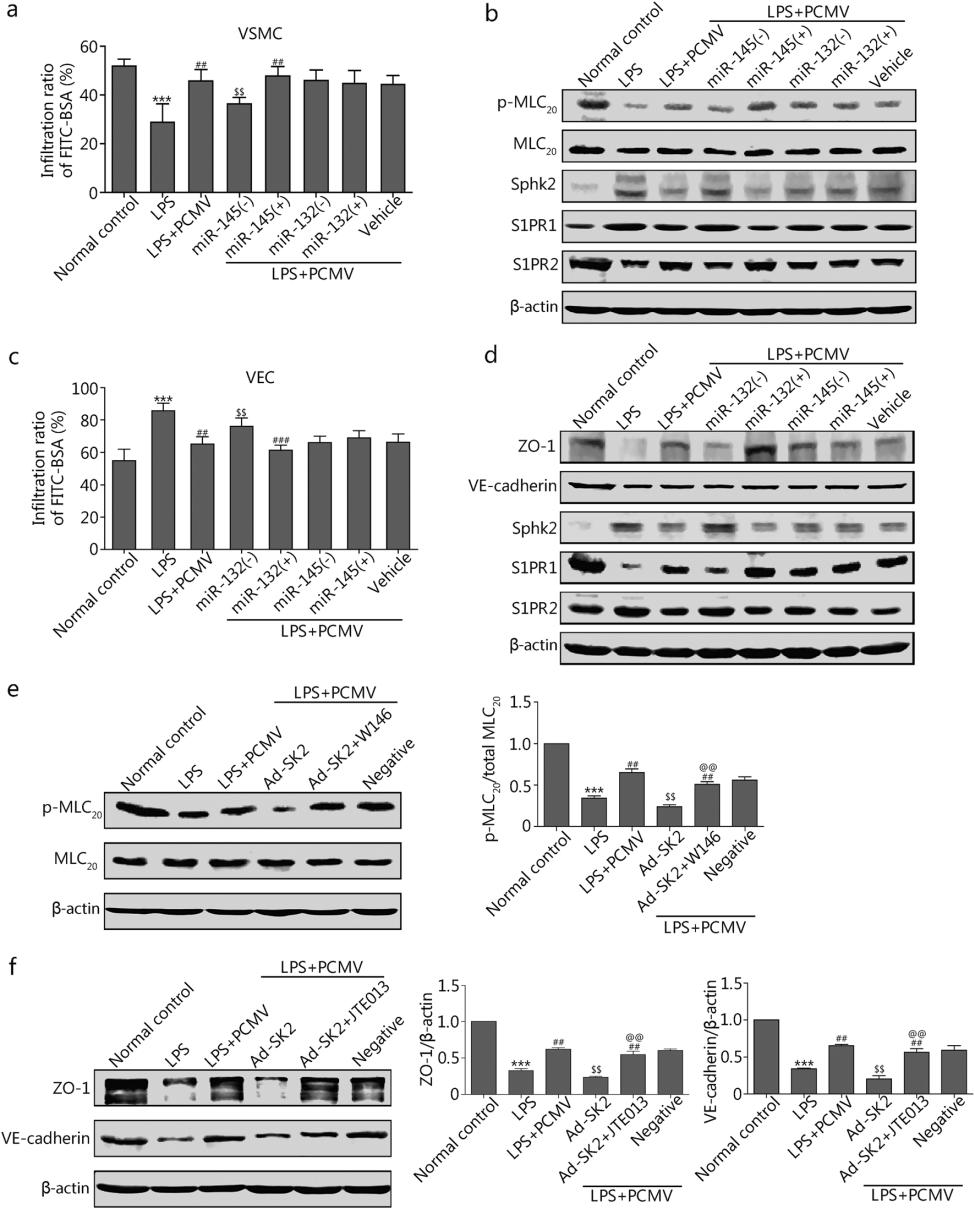
PCMVs carry miR-145 and miR-132 to play coordinated effects on the VSMCs and VECs.** a** Effects of different types of PCMVs on contractile response of rat VSMC to NE after LPS administration (*n* = 8 cells). **b** Western blotting analysis of p-MLC_20_, Sphk2, S1PR1 and S1PR2 from VSMCs treated with different types of PCMVs (*n* = 3 cells). **c** Different types of PCMVs were added into rat VECs, and FITC–BSA penetration of each group was measured (*n* = 8 cells).** d** Western blotting analysis of ZO-1, VE-cadherin, Sphk2, S1PR1 and S1PR2 from VECs treated with different types of PCMVs (*n* = 3 cells). **e–f** Western blotting analysis of p-MLC_20_, ZO-1 and VE-cadherin from VSMCs and VECs with S1PR1 inhibition (W146) and S1PR2 inhibition (JTE013) (*n* = 3 cells). LPS lipopolysaccharides, PC pericyte, PCMV pericyte-derived microvesicle, VECs vascular endothelial cells, VSMCs vascular smooth muscle cells, Sphk2 sphingosine kinase 2, S1PR1 sphingosine-1-phosphate receptor 1, p-MLC_20_ phosphorylation of myosin light chain 20, ZO-1 zonula occludens-1, VE-cadherin vascular endothelial cadherin, Ad-SK2 adenovirus-mediated overexpression of Sphk2, Negative infection of VSMCs or VECs with negative control adenovirus. Data shown as mean ± SD. ^***^P < 0.001 vs. Normal control; ^##^*P* < 0.01, ^###^*P* < 0.001 vs. LPS; ^$$^*P* < 0.01 vs. LPS + PCMV; ^@@^*P* < 0.01 vs. Ad-SK2 (one-way ANOVA)

To elucidate whether the protective effects of PCMVs on vascular function were attributable to miRNAs, we screened the main miRNAs related to VSMCs (miR-1, miR-15b, miR-143, miR-145, miR-147, and miR-503) and VECs (miR-23b, miR-125, miR-126, and miR-132) in PCMVs. miR-132 and miR-145 were more abundant in the PCMVs generated by pericytes and Poly(I:C)-treated pericytes (Additional file 1: Fig. S12a). To further determine whether miR-132 and miR-145 in PCMVs regulate the contractile response of VSMCs and barrier function of VECs, miR-145/132 inhibitors and mimics were used to obtain miR-145/132^downregulated^-PCMV [miR-145/132(-)PCMV] and miR-145/132^upregulated^-PCMV [miR-145/132(+)PCMV], respectively. Results showed that miRNA-145 and miRNA-132 acted on VSMCs and VECs to protect vascular reactivity and barrier function, respectively. In cultured VSMCs, miR-145(+)PCMV enhanced the effects of PCMVs on VSMCs (*P* < 0.01), whereas miR-145(-)PCMV attenuated the protective effect of PCMVs on VSMCs (*P* < 0.01). However, miR-132(−/+)PCMV had no effect on the constriction response of VSMCs (Fig. 5a, b; Additional file 1: Fig. S12b). In contrast, in cultured VECs, miR-132( +)PCMV enhanced the protective effect of PCMVs on the VEC barrier function (*P* < 0.001), whereas miR-132(−)PCMV attenuated VEC barrier function (*P* < 0.01). miR-145(−/+)PCMV did not affect VEC barrier function (Fig. 5c, d; Additional file 1: Fig. S12c-e).

Dual-luciferase reporter assay indicated that miR-132 and miR-145 acted on Sphk2 mRNA in VECs and VSMCs, respectively (Additional file 1: Fig. S12f). Briefly, the expression of Sphk2 was significantly increased upon LPS exposure. Experiments using Sphk2 overexpression in VSMCs and VECs, S1PR1 inhibitor W146 and S1PR2 inhibitor JTE013 suggested that high Sphk2 expression antagonized the protective effect of PCMVs, whereas S1PR1 inhibition restored the protective role of PCMVs on p-MLC_20_ in VSMCs. In VECs, overexpression of Sphk2 antagonized the protective effect of PCMVs, whereas inhibition of S1PR2 restored its endothelial barrier protective roles (Fig. 5e, f).

## Discussion

**Fig. 6:**
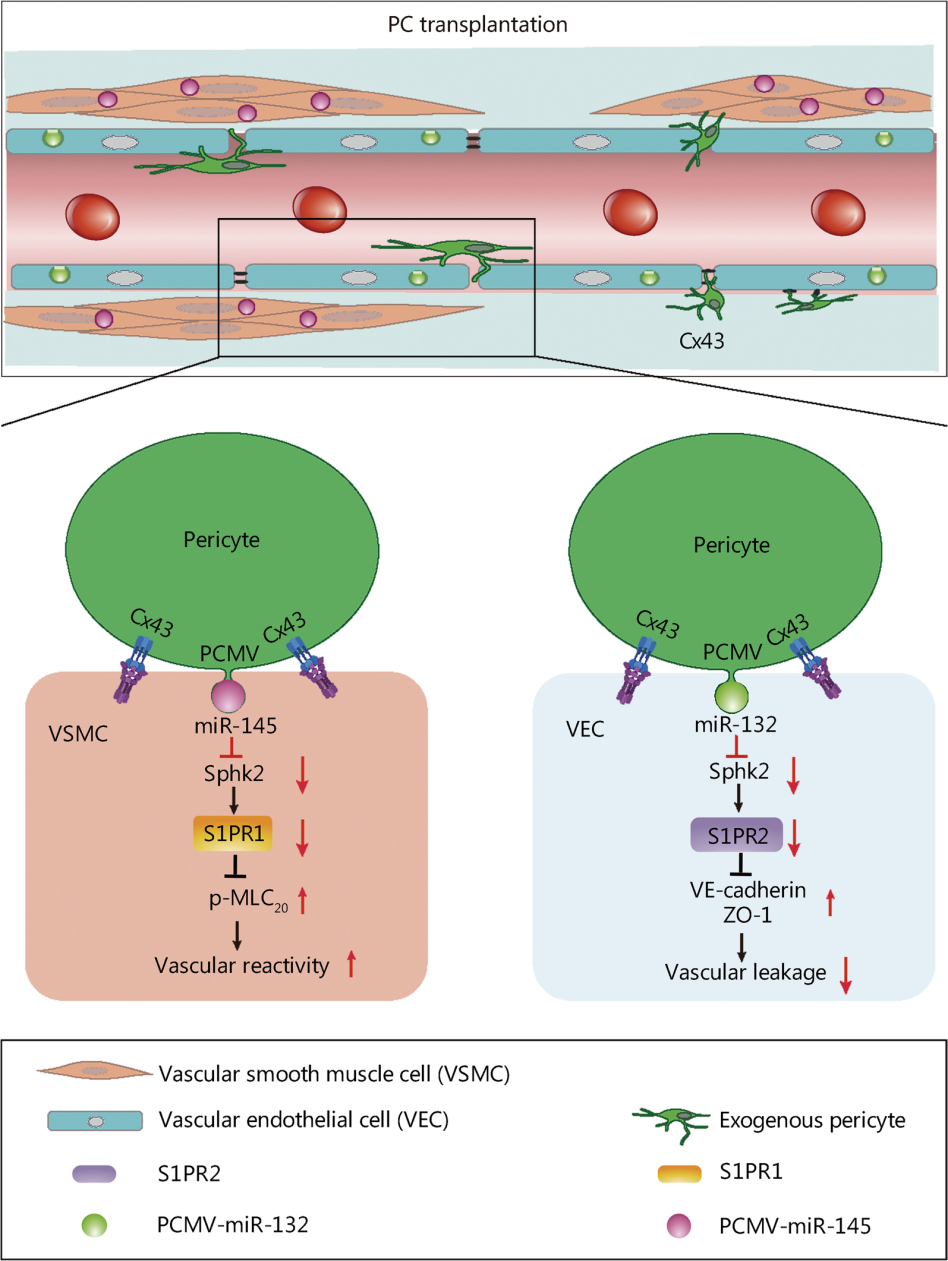
A schematic diagram of the protective role of pericytes in sepsis. After sepsis, pericyte desquamation, increased expression of endothelial S1PR2 and decreased ZO-1 and VE-cadherin are associated with vascular endothelial barrier breakdown; increased expression of S1PR1 and decreased p-MLC_20_ in VSMCs are associated with the vascular hyporeactivity. After pericyte transplantation, pericytes colonize in the mesenteric vein and form direct contact with endothelial cells to form a gap junction. Pericytes also secreted microvesicles (MVs) containing miR-145/132 to VSMCs and VECs to produce additional protective effects. miR-145 mainly acts on VSMCs to improve the vascular reactivity via inhibiting the expression of Sphk2 and S1PR1, and increasing the expression of p-MLC_20_. miR-132 mainly acts on VECs to improve the barrier function via inhibiting the expression of Sphk2 and S1PR2, and increasing the expression of ZO-1 and VE-cadherin. PC pericyte, PCMV pericyte-derived microvesicle, VEC vascular endothelial cell, VSMC vascular smooth muscle cell, p-MLC_20_ phosphorylation of myosin light chain 20, Sphk2 sphingosine kinase 2, S1PR1 sphingosine-1-phosphate receptor 1, ZO-1 zonula occludens-1, VE-cadherin vascular endothelial cadherin

Sepsis is defined as a life-threatening organ dysfunction caused by dysregulated host response to infection, with high morbidity and mortality rates [32]. Vascular hyporeactivity and leakage are key pathophysiologic features that cause MODS [2, 3]. A variety of treatment novel strategies have been proposed, but only a few have demonstrated sufficient therapeutic efficacy [33, 34]. Moreover, some of these strategies are inherently problematic. For example, vasoconstrictors (e.g., NE and arginine vasopressin) increase vascular reactivity but can also cause endothelial cytoskeleton contraction and subsequent aggravation of vascular leakage [33, 34]. Results from the current study indicate that pericyte transplantation can protect sepsis-induced vascular dysfunction by colonizing and covering microvessels and releasing microvesicles. Treatment with Poly(I:C)-treated pericytes elicited stronger effects than untreated pericytes. Importantly, Cx43 was found to play a crucial role in pericyte microvasculature colonization and coverage. miR-145 and miR-132 were identified as the key factors carried by PCMVs that contribute to the regulatory and protective roles in vascular contractile and barrier functions (Fig. 6).

Pericytes are pluripotent cells embedded in the vascular basement membrane and play key roles in the regeneration of microvessels and regulation of local blood flow. However, pericytes also contribute to the regulation of contractile and angiogenic functions, exhibit immune-promoting properties, and regenerate the cell types that constitute the tissue in which they exist [7, 8]. Pericyte transplantation thus could facilitate tissue repair, including the skeletal muscle, heart and bone tissues, after injury. For instance, Munroe et al. [35] reported that pericyte transplantation improved skeletal muscle recovery following hindlimb immobilization. Alvino et al. [36] observed that allogeneic pericytes improved myocardial vascularization and reduced interstitial fibrosis in a swine model of acute myocardial infarction. Konig et al. [37] found that transplantation of pericytes from adipose tissue promoted the healing of critical-sized bone defects. However, no previous studies have reported whether pericytes are protective against sepsis-induced vascular dysfunction. In the current study, we found that sepsis damaged endogenous pericytes in microvessels and that loss or chemical depletion of pericytes exacerbated vascular hyporeactivity and leakage. Pericyte transplantation protected both rats and mice from sepsis-induced vascular hyporeactivity and vascular leakage.

Pericytes are connected to endothelial cells by several types of intercellular junctions, including tight junctions, gap junctions, and adhesion plaques [13]. In particular, gap junctions (Cx43/30) play important roles in the connection of pericytes and VECs and allow direct communication between neighboring cells via diffusion of nutrients, metabolites, secondary messengers, ions and various other molecules [38]. The current study suggested a new mechanism by which Cx43 regulates the colonization of transplanted pericytes, and extended our current knowledge beyond the traditional role of Cx43 in direct cell–cell communication through gap junctions [39]. We hypothesized that transplanted pericytes could form gap junction via Cx43 and promote endothelial barrier function.

Optimal colonization of pericytes was observed 24 h after transplantation. However, improved vascular function was detected as early as 6 h, suggesting additional mechanisms that are not dependent on pericyte colonization. Indeed, PCMVs exerted protective effects primarily by transferring miRNA-145 and miRNA-132 to VSMCs and VECs, respectively. miR-145 and miR-132 have important roles in vascular leakage, maturation and reparative angiogenesis [40, 41]. In particular, miR-145 is highly enriched in VSMCs and promotes the contractile phenotype, thus playing a crucial role in several cardiovascular diseases, including hypertension and coronary artery disease [42]. miR-132 is highly conserved and abundantly expressed in normal VECs, where it regulates VEC proliferation and migration. miR-132 also plays a key role in promoting VEC angiogenesis and maintaining vascular integrity [43]. The current study suggested that pericytes may secrete PCMVs and transfer miR-145 and miR-132 to VSMCs and VECs to protect vascular reactivity and vascular barrier function in septic rats and mice.

The protective effects elicited by PCMV-associated miR-145 and miR-132 could involve the Sphk2/S1PR1/p-MLC_20_ pathway in VSMCs and Sphk2/S1PR2/ ZO-1 and VE-cadherin pathway in VECs, respectively. The S1P signaling pathway plays a key role in regulating endothelial barrier function and angiogenesis via the G protein-coupled receptors S1PR1 and S1PR2 [44]. S1P can also counteract pericyte loss and microvessel disassembly during sepsis [45]. S1PR1 was originally identified as an abundant transcript in endothelial cells that contributes to the regulation of endothelial cell cytoskeletal structure, migration, capillary-like network formation, and vascular maturation [46]. S1PR2 is highly expressed in neuronal cells and VSMCs and has been implicated in various biological processes, including cell migration, contraction and differentiation, by regulating the expression of smooth muscle differentiation genes [47]. Under normal conditions, S1PR1 and S1PR2 are primarily expressed in VECs and VSMCs, respectively. S1PR2 expression is increased in VECs where it aggravates vascular permeability upon inflammation; S1PR1 is increased in VSMCs and decreased in vascular reactivity in cardiovascular disorders [48]. Sphk2 catalyzes the phosphorylation of sphingosine to S1P, and participates in inflammation, endoplasmic reticulum stress, and apoptosis [49]. We found increased expression of both Sphk2 and S1PR1 in VSMCs upon LPS exposure. miR-145 released by PCMVs inhibited Sphk2 and S1PR1, and restored the contractile function of VSMCs by upregulating p-MLC_20_. In contrast, miR-132 released by PCMVs inhibited Sphk2 and S1PR2, and restored the barrier function of VECs by upregulating ZO-1 and VE-cadherin expression.

Poly(I:C), a dsRNA analog and inducer of interferon, has broad-spectrum antiviral and immunoregulatory effects. Mesenchymal stem cell (MSC)-based therapy is a promising approach for many critical diseases, such as graft-versus-host disease, autoimmune diseases, and kidney, liver, and heart injury, owing to their prominent ability in immune regulation [50]. Poly(I:C) has been shown to reduce the immunogenicity of MSCs and enhance their paracrine functions [51]. In animal models of CLP-induced sepsis, Poly(I:C) improves the immunosuppressive properties of MSCs and animal survival by inhibiting miR-143 [52]. Poly(I:C)-pretreated MSCs also enhance the antimicrobial effects of microvesicles in injured lungs [53]. In the current study, Poly(I:C)-stimulated pericytes produced higher amount of PCMVs with higher levels of miR-145 and miR-132 and elicited superior protective effects on vascular reactivity and barrier function following sepsis.

The current study has several limitations. First, pericytes are broadly distributed but only mesenteric microvessels were examined in this study. Second, we only assessed the role of LPS and CLP in pericyte damage and loss without elucidating the mechanisms underlying LPS- and CLP-induced pericyte damage and loss. Third, although the relationship between Cx43 and pericyte colonization was clearly demonstrated, the underlying mechanisms remain unknown. Fourth, miR-145 and miR-132 could be produced by cells other than VECs and VSMCs; whether endogenous miR-145 and miR-132 from other sources also contribute to the protective action of pericytes in sepsis requires further investigation. Fifth, considering the high mortality of PDGFR-β knockout mice, we used CP-673451, a PDGFR-β inhibitor, to deplete pericytes based on a previously published protocol [20]. Importantly, we found that pericytes in the mesenteric microvascular networks were successfully depleted by 7 d of CP-673451 treatment without noticeably damaging the function of other vital organs. Prolonged treatment, however, produced significant effects beyond the mesenteric microvascular networks. Also, the extent of pericytes depletion with CP-673451 treatment was not complete. Finally, although two control groups (a sepsis control and a conventional treatment control) were included, all experiments were conducted in rodents. Further investigation with large animals (e.g., non-human primates) are warranted.

## Conclusions

In summary, the present study showed that pericyte loss and structural destruction contribute to vascular hyporeactivity and leakage upon sepsis. Pericyte transplantation could protect the contractile and barrier functions of vasculature via colonization and direct coverage as well as PCMV release.

## Supplementary Information

**Additional file 1. Fig. S1** Production of PDGFR-β-Cre + mT/mG transgenic mice and Tie2-Cre + Cx43flox/flox mice. **Fig. S2** Sepsis induces pericyte loss, vascular hyporeactivity and leakage. **Fig. S3** Sepsis induces pericyte loss, vascular hyporeactivity and leakage in PDGFR-β-Cre + mT/mG transgenic and WT mice. **Fig. S4** Primary pericyte identification, the number of pericyte colonization at different times, and the effect of pericyte transplantation on vascular reactivity and barrier function in septic rats within 6 h. **Fig. S5** Pericyte transplantation increases the pericyte coverage and improves the vascular functions at 24 h after sepsis. **Fig. S6** Effect of Cx43 on vascular reactivity and permeability of VSMCs/VECs through Cx43. **Fig. S7** Depletion of pericytes with CP-673451. **Fig. S8** Pericyte depletion aggravates sepsis-induced pericyte loss, which in turn is rescued by pericyte transplantation. **Fig. S9** Pericyte depletion aggravates sepsis-induced vascular hyporeactivity, which in turn is rescued by pericyte transplantation. **Fig. S10** Pericyte depletion aggravates sepsis-induced vascular leakage, which in turn is rescued by pericyte transplantation. **Fig. S11** PCMVs improve the contractile response of VSMCs and barrier function of VECs after sepsis. **Fig. S12** PCMVs carry miR-145 and miR-132 to VSMCs and VECs to play orchestrate effects on the contractile response of VSMCs and barrier function of VECs.

**Additional file 2. Video S1**: Vascualr reactivity of sham group.

**Additional file 3. Video S2**: PC VSMC.

**Additional file 4. Video S3**: PC Cx43 down VSMC.

**Additional file 5. Video S4**: PC vehicle VSMC.

**Additional file 6. Video S5**: PC VEC.

**Additional file 7. Video S6**: PC Cx43 down VEC.

**Additional file 8. Video S7**: PC vehicle VEC.

## Data Availability

All data generated or analyzed during this study are included in this published article.

## Abbreviations

Ach: Acetylcholine
CLP: Cecal ligation and puncture
Cx43: Connexin 43
CT: Conventional treatment
CLSM: Confocal laser scanning microscopy
DLS: Dynamic light scattering
GFP: Green fluorescent protein
H/S: Hypoxia/starvation
IV: Intensity in the venules
IP: Intensity in the perivenular interstitium
LPS: Lipopolysaccharide
MA: Mesenteric arteriole
MODS: Multiple organ dysfunction syndrome
NE: Norepinephrine
NG-2: Nerve/glial antigen 2
PCMV: PC-derived microvesicle
p-MLC_20_: Phosphorylation of myosin light chain 20
PDGFR-β: Platelet-derived growth factor receptor beta
RBC: Red blood cell
SEM: Scanning electron microscopy
Sphk2: Sphingosine kinase 2
S1PR1: Sphingosine-1-phosphate receptor 1
SMV: Superior mesenteric vein
SMA: Superior mesenteric artery
TEM: Transmission electron microscopy
TJ: Tight junction
TEER: Transendothelial electrical resistance
VEC: Vascular endothelial cell
VSMC: Vascular smooth muscle cell
VE-cadherin: Vascular endothelial cadherin
ZO-1: Zonula occludens-1
α-SMA: α-Smooth muscle actin

## References

[1] Yao YM, Zhang H (2019). Better therapy for combat injury. Mil Med Res.

[2] Singh V, Akash R, Chaudhary G, Singh R, Choudhury S, Shukla A (2022). Sepsis downregulates aortic Notch signaling to produce vascular hyporeactivity in mice. Sci Rep.

[3] Ince C, Mayeux PR, Nguyen T, Gomez H, Kellum JA, Ospina-Tascon GA (2016). The endothelium in sepsis. Shock.

[4] Auriemma CL, Zhuo H, Delucchi K, Deiss T, Liu T, Jauregui A (2020). Acute respiratory distress syndrome-attributable mortality in critically ill patients with sepsis. Intensive Care Med.

[5] Senatore F, Balakumar P, Jagadeesh G (2021). Dysregulation of the renin-angiotensin system in septic shock: mechanistic insights and application of angiotensin II in clinical management. Pharmacol Res.

[6] Gauer R, Forbes D, Boyer N (2020). Sepsis: diagnosis and management. Am Fam Physician.

[7] Dalkara T (2019). Pericytes: a novel target to improve success of recanalization therapies. Stroke.

[8] Litvinukova M, Talavera-Lopez C, Maatz H, Reichart D, Worth CL, Lindberg EL (2020). Cells of the adult human heart. Nature.

[9] Meng YM, Jiang X, Zhao X, Meng Q, Wu S, Chen Y (2021). Hexokinase 2-driven glycolysis in pericytes activates their contractility leading to tumor blood vessel abnormalities. Nat Commun.

[10] Park SW, Yun JH, Kim JH, Kim KW, Cho CH, Kim JH (2014). Angiopoietin 2 induces pericyte apoptosis via α3β1 integrin signaling in diabetic retinopathy. Diabetes.

[11] Yuan K, Agarwal S, Chakraborty A, Condon DF, Patel H, Zhang S (2021). Lung pericytes in pulmonary vascular physiology and pathophysiology. Compr Physiol.

[12] Castro Dias M, Mapunda JA, Vladymyrov M, Engelhardt B (2019). Structure and junctional complexes of endothelial, epithelial and glial brain barriers. Int J Mol Sci.

[13] Ayloo S, Lazo CG, Sun S, Zhang W, Cui B, Gu C (2022). Pericyte-to-endothelial cell signaling via vitronectin-integrin regulates blood-CNS barrier. Neuron.

[14] Al Ahmad A, Taboada CB, Gassmann M, Ogunshola OO (2011). Astrocytes and pericytes differentially modulate blood-brain barrier characteristics during development and hypoxic insult. J Cereb Blood Flow Metab.

[15] Liu C, Ge HM, Liu BH, Dong R, Shan K, Chen X (2019). Targeting pericyte-endothelial cell crosstalk by circular RNA-cPWWP2A inhibition aggravates diabetes-induced microvascular dysfunction. Proc Natl Acad Sci U S A.

[16] Avolio E, Katare R, Thomas AC, Caporali A, Schwenke D, Carrabba M (2022). Cardiac pericyte reprogramming by MEK inhibition promotes arteriologenesis and angiogenesis of the ischemic heart. J Clin Invest.

[17] Kisler K, Nelson AR, Rege SV, Ramanathan A, Wang Y, Ahuja A (2017). Pericyte degeneration leads to neurovascular uncoupling and limits oxygen supply to brain. Nat Neurosci.

[18] Abed A, Toubas J, Kavvadas P, Authier F, Cathelin D, Alfieri C (2014). Targeting connexin 43 protects against the progression of experimental chronic kidney disease in mice. Kidney Int.

[19] Nishii K, Seki A, Kumai M, Morimoto S, Miwa T, Hagiwara N (2016). Connexin45 contributes to global cardiovascular development by establishing myocardial impulse propagation. Mech Dev.

[20] Chintalgattu V, Rees ML, Culver JC, Goel A, Jiffar T, Zhang J (2013). Coronary microvascular pericytes are the cellular target of sunitinib malate-induced cardiotoxicity. Sci Transl Med..

[21] Rittirsch D, Huber-Lang MS, Flierl MA, Ward PA (2009). Immunodesign of experimental sepsis by cecal ligation and puncture. Nat Protoc.

[22] Nishiguchi T, Cho K, Isaka S, Ueno M, Jin JO, Yamaguchi K (2016). Protective effect of porphyran isolated from discolored nori (*Porphyra yezoensis*) on lipopolysaccharide-induced endotoxin shock in mice. Int J Biol Macromol.

[23] Liu G, Meng C, Pan M, Chen M, Deng R, Lin L (2014). Isolation, purification, and cultivation of primary retinal microvascular pericytes: a novel model using rats. Microcirculation.

[24] Duan C, Wang L, Zhang J, Xiang X, Wu Y, Zhang Z (2020). Mdivi-1 attenuates oxidative stress and exerts vascular protection in ischemic/hypoxic injury by a mechanism independent of Drp1 GTPase activity. Redox Biol.

[25] Gaceb A, Ozen I, Padel T, Barbariga M, Paul G (2018). Pericytes secrete pro-regenerative molecules in response to platelet-derived growth factor-BB. J Cereb Blood Flow Metab.

[26] Jung MK, Mun JY (2018). Sample preparation and imaging of exosomes by transmission electron microscopy. J Vis Exp.

[27] Wang Y, Zhang L, Li Y, Chen L, Wang X, Guo W (2015). Exosomes/microvesicles from induced pluripotent stem cells deliver cardioprotective miRNAs and prevent cardiomyocyte apoptosis in the ischemic myocardium. Int J Cardiol.

[28] Zhang J, Yang G, Zhu Y, Peng X, Li T, Liu L (2018). Relationship of Cx43 regulation of vascular permeability to osteopontin-tight junction protein pathway after sepsis in rats. Am J Physiol Regul Integr Comp Physiol.

[29] Yang G, Li T, Xu J, Liu L (2010). PKC plays an important mediated effect in arginine vasopressin induced restoration of vascular responsiveness and calcium sensitization following hemorrhagic shock in rats. Eur J Pharmacol.

[30] Jing H, Zhang X, Luo K, Luo Q, Yin M, Wang W (2020). miR-381-abundant small extracellular vesicles derived from kartogenin-preconditioned mesenchymal stem cells promote chondrogenesis of MSCs by targeting TAOK1. Biomaterials.

[31] Zhou H, Zheng D, Wang H, Wu Y, Peng X, Li Q (2021). The protective effects of pericyte-derived microvesicles on vascular endothelial functions via CTGF delivery in sepsis. Cell Commun Signal.

[32] Singer M, Deutschman CS, Seymour CW, Shankar-Hari M, Annane D, Bauer M (2016). The third international consensus definitions for sepsis and septic shock (Sepsis-3). JAMA.

[33] Li Z, Yin M, Zhang H, Ni W, Pierce RW, Zhou HJ (2020). BMX represses thrombin-PAR1-mediated endothelial permeability and vascular leakage during early sepsis. Circ Res.

[34] Joffre J, Hellman J, Ince C, Ait-Oufella H (2020). Endothelial responses in sepsis. Am J Respir Crit Care Med.

[35] Munroe M, Dvoretskiy S, Lopez A, Leong J, Dyle MC, Kong H (2019). Pericyte transplantation improves skeletal muscle recovery following hindlimb immobilization. FASEB J.

[36] Alvino VV, Fernández-Jiménez R, Rodriguez-Arabaolaza I, Slater S, Mangialardi G, Avolio E (2018). Transplantation of allogeneic pericytes improves myocardial vascularization and reduces interstitial fibrosis in a swine model of reperfused acute myocardial infarction. J Am Heart Assoc.

[37] Konig MA, Canepa DD, Cadosch D, Casanova E, Heinzelmann M, Rittirsch D (2016). Direct transplantation of native pericytes from adipose tissue: a new perspective to stimulate healing in critical size bone defects. Cytotherapy.

[38] Alarcon-Martinez L, Villafranca-Baughman D, Quintero H, Kacerovsky JB, Dotigny F, Murai KK (2020). Interpericyte tunnelling nanotubes regulate neurovascular coupling. Nature.

[39] Medina-Flores F, Hurtado-Alvarado G, Contis-Montes De Oca A, Lopez-Cervantes SP, Konigsberg M, Deli MA (2020). Sleep loss disrupts pericyte-brain endothelial cell interactions impairing blood–brain barrier function. Brain Behav Immun.

[40] Katare R, Riu F, Mitchell K, Gubernator M, Campagnolo P, Cui Y (2011). Transplantation of human pericyte progenitor cells improves the repair of infarcted heart through activation of an angiogenic program involving micro-RNA-132. Circ Res.

[41] Wu Y, Li P, Goodwin AJ, Cook JA, Halushka PV, Zingarelli B (2020). miR-145a regulation of pericyte dysfunction in a murine model of sepsis. J Infect Dis.

[42] Chin DD, Poon C, Wang J, Joo J, Ong V, Jiang Z (2021). miR-145 micelles mitigate atherosclerosis by modulating vascular smooth muscle cell phenotype. Biomaterials.

[43] Xu B, Zhang Y, Du XF, Li J, Zi HX, Bu JW (2017). Neurons secrete miR-132-containing exosomes to regulate brain vascular integrity. Cell Res.

[44] Cartier A, Leigh T, Liu CH, Hla T (2020). Endothelial sphingosine 1-phosphate receptors promote vascular normalization and antitumor therapy. Proc Natl Acad Sci U S A.

[45] Abdel Rahman F, D'almeida S, Zhang T, Asadi M, Bozoglu T, Bongiovanni D (2021). Sphingosine-1-phosphate attenuates lipopolysaccharide-induced pericyte loss via activation of Rho-A and MRTF-A. Thromb Haemost.

[46] Hubner K, Cabochette P, Dieguez-Hurtado R, Wiesner C, Wakayama Y, Grassme KS (2018). Wnt/β-catenin signaling regulates VE-cadherin-mediated anastomosis of brain capillaries by counteracting S1pr1 signaling. Nat Commun.

[47] Hoefer J, Azam MA, Kroetsch JT, Leong-Poi H, Momen MA, Voigtlaender-Bolz J (2010). Sphingosine-1-phosphate-dependent activation of p38 MAPK maintains elevated peripheral resistance in heart failure through increased myogenic vasoconstriction. Circ Res.

[48] Lee JF, Gordon S, Estrada R, Wang L, Siow DL, Wattenberg BW (2009). Balance of S1P1 and S1P2 signaling regulates peripheral microvascular permeability in rat cremaster muscle vasculature. Am J Physiol Heart Circ Physiol.

[49] Park SJ, Kim JM, Kim J, Hur J, Park S, Kim K (2018). Molecular mechanisms of biogenesis of apoptotic exosome-like vesicles and their roles as damage-associated molecular patterns. Proc Natl Acad Sci U S A.

[50] Deng L, Li H, Su X, Zhang Y, Xu H, Fan L (2020). Chlorzoxazone, a small molecule drug, augments immunosuppressive capacity of mesenchymal stem cells via modulation of FOXO3 phosphorylation. Cell Death Dis.

[51] Evaristo-Mendonca F, Sardella-Silva G, Kasai-Brunswick TH, Campos RMP, Domizi P, Santiago MF (2019). Preconditioning of rat bone marrow-derived mesenchymal stromal cells with Toll-like receptor agonists. Stem Cells Int.

[52] Zhao X, Liu D, Gong W, Zhao G, Liu L, Yang L (2014). The toll-like receptor 3 ligand, poly(I:C), improves immunosuppressive function and therapeutic effect of mesenchymal stem cells on sepsis via inhibiting MiR-143. Stem Cells.

[53] Park J, Kim S, Lim H, Liu A, Hu S, Lee J (2019). Therapeutic effects of human mesenchymal stem cell microvesicles in an ex vivo perfused human lung injured with severe *E. coli* pneumonia. Thorax.

