# Supplementary material for: Pericytes protect rats and mice from sepsis-induced injuries by maintaining vascular reactivity and barrier function: implication of miRNAs and microvesicles
Source: Mil Med Res. 2023 Mar 13;10:13. doi: 10.1186/s40779-023-00442-2 (PMC10010010; doi:10.1186/s40779-023-00442-2)
Supplement: Supplementary file 1 — Additional file 1. Fig. S1 Production of PDGFR-β-Cre + mT/mG transgenic mice and Tie2-Cre + Cx43flox/flox mice. Fig. S2 Sepsis induces pericyte loss, vascular hyporeactivity and leakage. Fig. S3 Sepsis induces pericyte loss, vascular hyporeactivity and leakage in PDGFR-β-Cre + mT/mG transgenic and WT mice. Fig. S4 Primary pericyte identification, the number of pericyte colonization at different times, and the effect of pericyte transplantation on vascular reactivity and barrier function in septic rats within 6 h. Fig. S5 Pericyte transplantation increases the pericyte coverage and improves the vascular functions at 24 h after sepsis. Fig. S6 Effect of Cx43 on vascular reactivity and permeability of VSMCs/VECs through Cx43. Fig. S7 Depletion of pericytes with CP-673451. Fig. S8 Pericyte depletion aggravates sepsis-induced pericyte loss, which in turn is rescued by pericyte transplantation. Fig. S9 Pericyte depletion aggravates sepsis-induced vascular hyporeactivity, which in turn is rescued by pericyte transplantation. Fig. S10 Pericyte depletion aggravates sepsis-induced vascular leakage, which in turn is rescued by pericyte transplantation. Fig. S11 PCMVs improve the contractile response of VSMCs and barrier function of VECs after sepsis. Fig. S12 PCMVs carry miR-145 and miR-132 to VSMCs and VECs to play orchestrate effects on the contractile response of VSMCs and barrier function of VECs. [file 40779_2023_442_MOESM1_ESM.pdf]

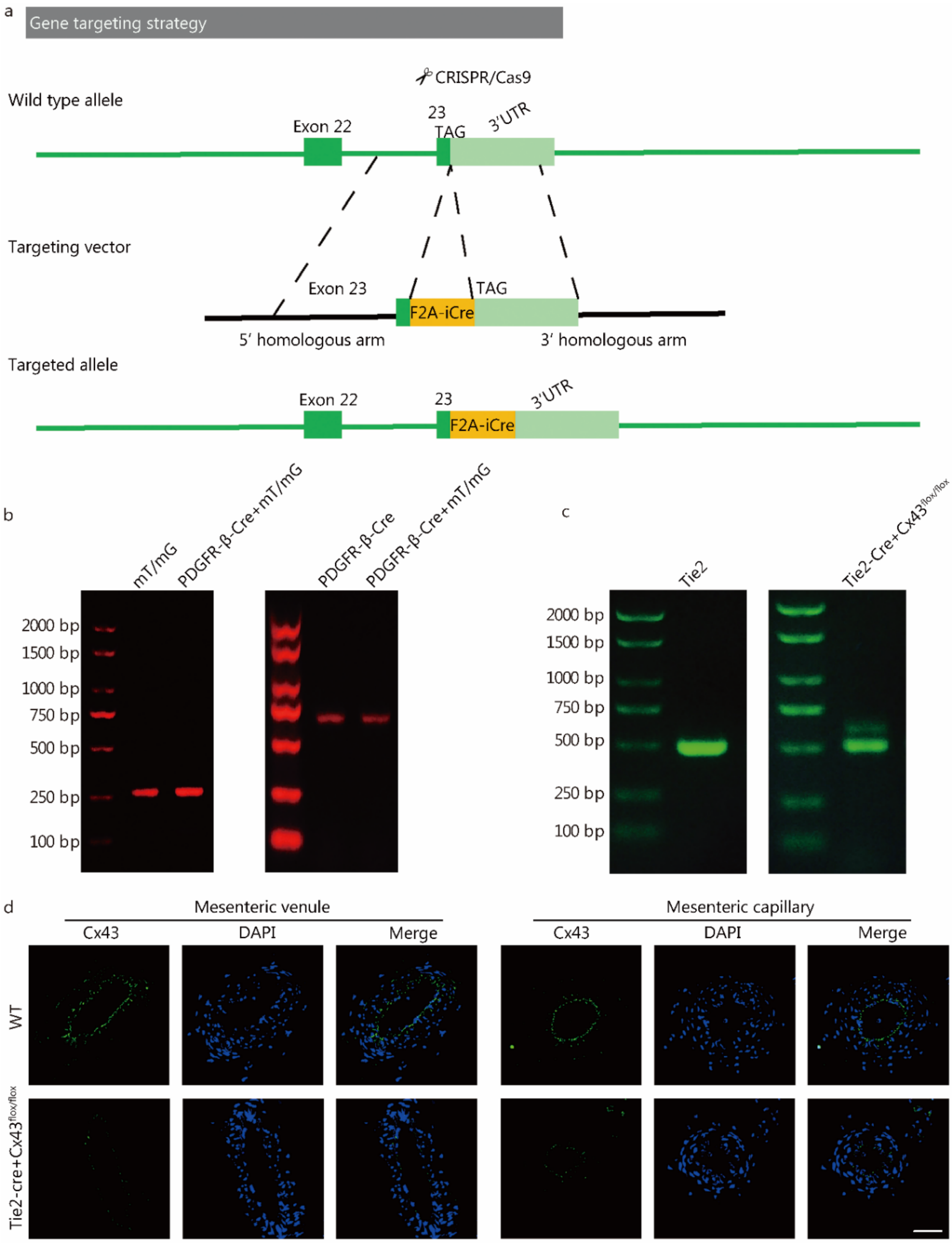

Fig. S1 Production of PDGFR-β-Cre + mT/mG transgenic mice and Tie2-Cre + Cx43$^{flox/flox}$ mice. **a-c** Generation and characterization of PDGFR-β-Cre + mT/mG transgenic mice and Tie2-Cre + Cx43$^{flox/flox}$ mice. **d** Mesenteric venule and capillary from WT and Tie2-Cre + Cx43$^{flox/flox}$ mice were stained with an antibody against Cx43. Scale bars: 50 μm. TAG terminal codon, 3'UTR three prime untranslated region, mT/mG membrane-Tomato before Cre/membrane-GFP after Cre, PDGFR-β platelet-derived growth factor receptor beta, Cre cyclization recombination enzyme, WT wide type

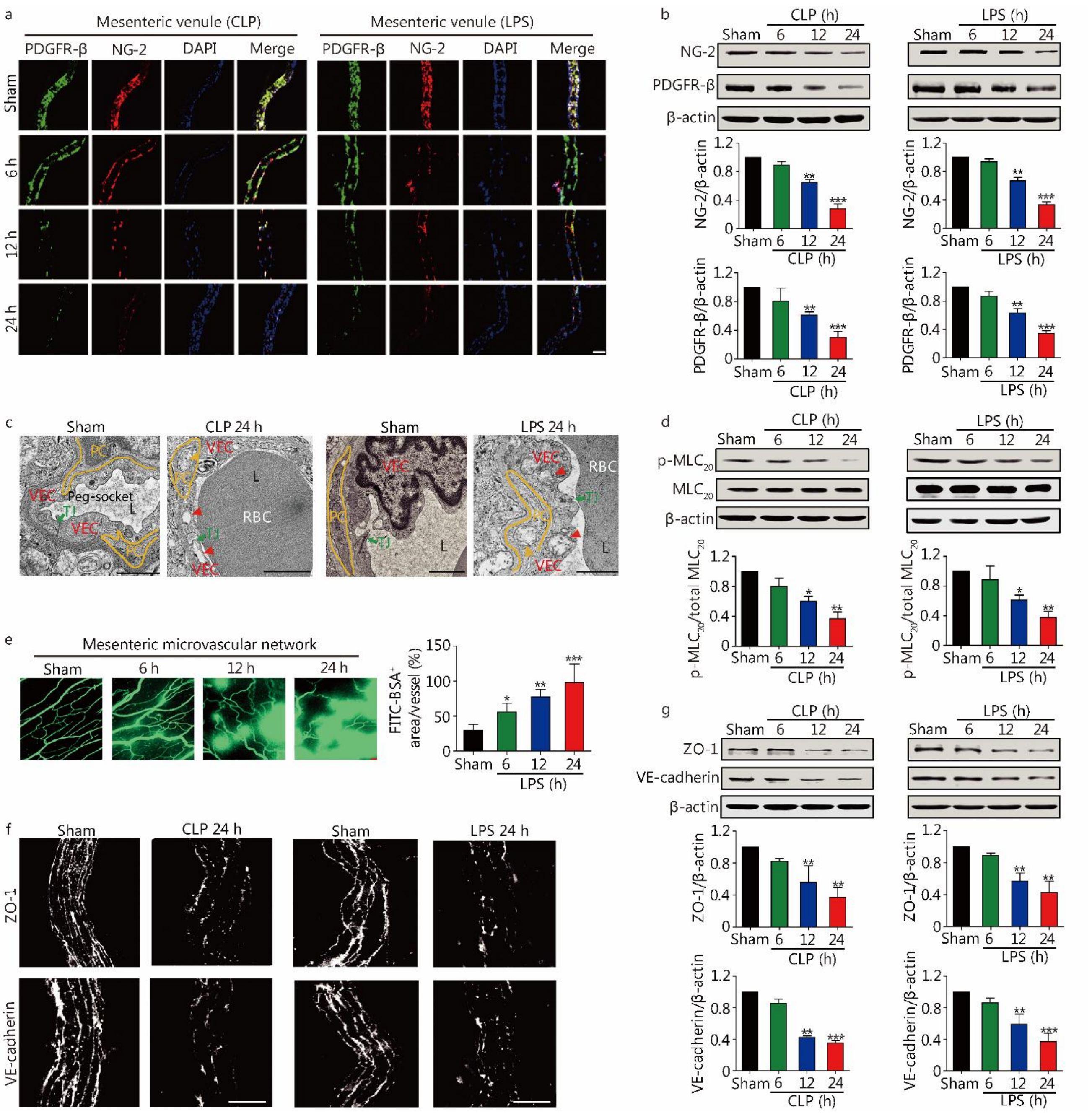

Fig. S2 Sepsis induces pericyte loss, vascular hyporeactivity and leakage. **a** Mesenteric venules from sham-operated rats and septic rats were stained with antibodies against pericytes markers (PDGFR-β, green; NG-2, red). Scale bars: 50 μm. **b** Western blotting analysis of NG-2 and PDGFR-β from mesenteric microvascular networks treated with CLP or LPS (*n* = 3 rats). **c** Representative TEM image of retina from sham-operated and septic rats at 24 h (green arrows indicate the tight junction, yellow and red arrowheads indicate pericyte and VEC swelling). Scale bars: 1 μm. **d** Expression of p-MLC_{20} and MLC_{20} in SMA of rats treated with CLP or LPS (*n* = 3 rats). **e** Vascular leakage of mesenteric microvascular networks measured by the appearance of intravenously injected of FITC–BSA and

quantitation of FITC–BSA$^+$ vessel (n = 8 rats). Scale bars: 50 μm. **f** Immunohistochemistry for ZO-1 and VE-cadherin in rat mesenteric venules. Scale bars: 20 μm. **g** Expression of ZO-1 and VE-cadherin in SMVs of rats treated with CLP or LPS (n = 3 rats). CLP cecal ligation and puncture, LPS lipopolysaccharides, PC pericyte, NG-2 nerve/glial antigen 2, PDGFR-β platelet-derived growth factor receptor beta, VEC vascular endothelial cell, RBC red blood cell, TJ tight junction, L lumen, p-MLC$_{20}$ phosphorylation of myosin light chain 20, SMA superior mesenteric artery, ZO-1 zonula occludens-1, VE-cadherin vascular endothelial cadherin, SMV superior mesenteric vein, TEM transmission electron microscopy. Data shown as mean ± SD. $^{*}P < 0.05$, $^{**}P < 0.01$, $^{***}P < 0.001$ vs. Sham (one-way ANOVA)

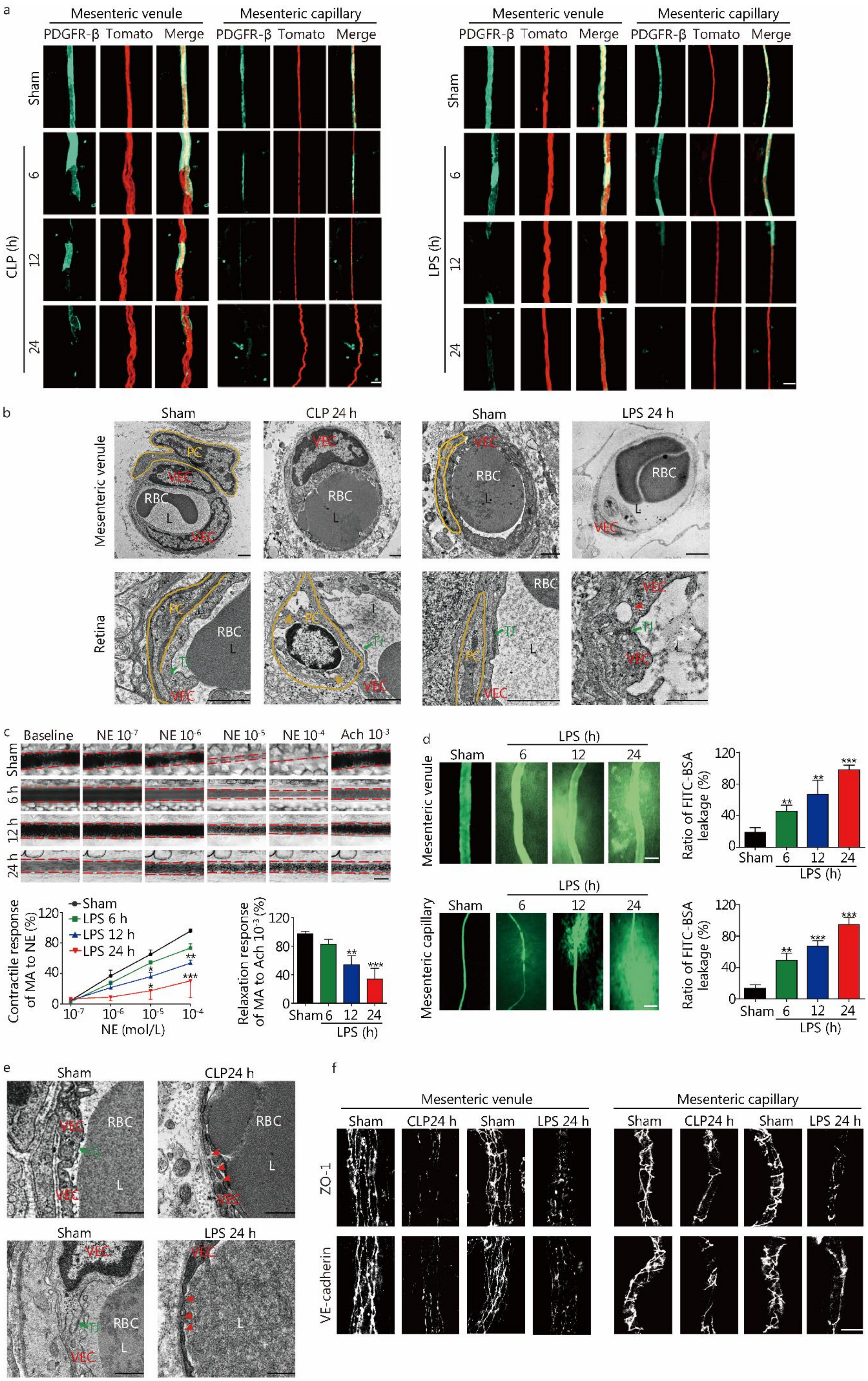

a
Mesenteric venule
Mesenteric capillary
PDGFR-β Tomato Merge
Sham
CLP (h)
6
12
24
LPS (h)
b
Sham
CLP 24 h
LPS 24 h
Mesenteric venule
Retina
PC
VEC
RBC
L
TJ
c
Baseline
NE 10-7
NE 10-6
NE 10-5
NE 10-4
Ach 10-3
Sham
6 h
12 h
24 h
Contractile response of MA to NE (%)
NE (mol/L)
LPS 6 h
LPS 12 h
LPS 24 h
Relaxation response of MA to Ach 10-3 (%)
d
Mesenteric venule
Mesenteric capillary
Ratio of FITC-BSA leakage (%)
e
CLP24 h
f
ZO-1
VE-cadherin

Fig. S3 Sepsis induces pericyte loss, vascular hyporeactivity and leakage in PDGFR-β-Cre + mT/mG transgenic and WT mice. **a** In vivo image of the mesenteric venules and capillaries of an PDGFR-β-Cre + mT/mG transgenic mice showing mGFP (green)-expressing pericytes on mesenteric microvessel with Tomato (red) expression in all membranes from the CLP- and LPS-induced sepsis at 6, 12, 24 h. Scale bars: 20 μm. **b** Representative TEM images of pericytes in mesenteric venules and retina after CLP or LPS at 24 h (green arrows indicate the tight junction, yellow arrowheads indicate pericyte swelling, and red arrowheads indicate VEC swelling). Scale bars: 1 μm. **c** Changes in vascular response of mesenteric arterioles (n = 8 mice). Scale bars: 50 μm. **d** Vascular leakage of mesenteric venules and capillaries measured by the appearance of intravenously injected of FITC–BSA and quantitation of FITC–BSA^{+} vessel (n = 8 mice). Scale bars: 20 μm. **e** Representative TEM images of tight junctions in mesenteric venules after CLP or LPS at 24 h (green arrows indicate the tight junction, red arrowheads indicate the disrupted VECs). Scale bars: 1 μm. **f** Immunohistochemistry for ZO-1 and VE-cadherin in mouse mesenteric venules and capillaries. Scale bars: 20 μm. PC pericyte, PDGFR-β platelet-derived growth factor receptor beta, CLP cecal ligation and puncture, LPS lipopolysaccharides, TEM transmission electron microscopy, NE norepinephrine, Ach acetylcholine, MA mesenteric arteriole, VEC vascular endothelial cell, RBC red blood cell, TJ tight junction, L lumen, ZO-1 zonula occludens-1, VE-cadherin vascular endothelial cadherin. Data shown as mean ± SD. $^{*}P < 0.05$, $^{**}P < 0.01$, $^{***}P < 0.001$ vs. Sham (one-way ANOVA)

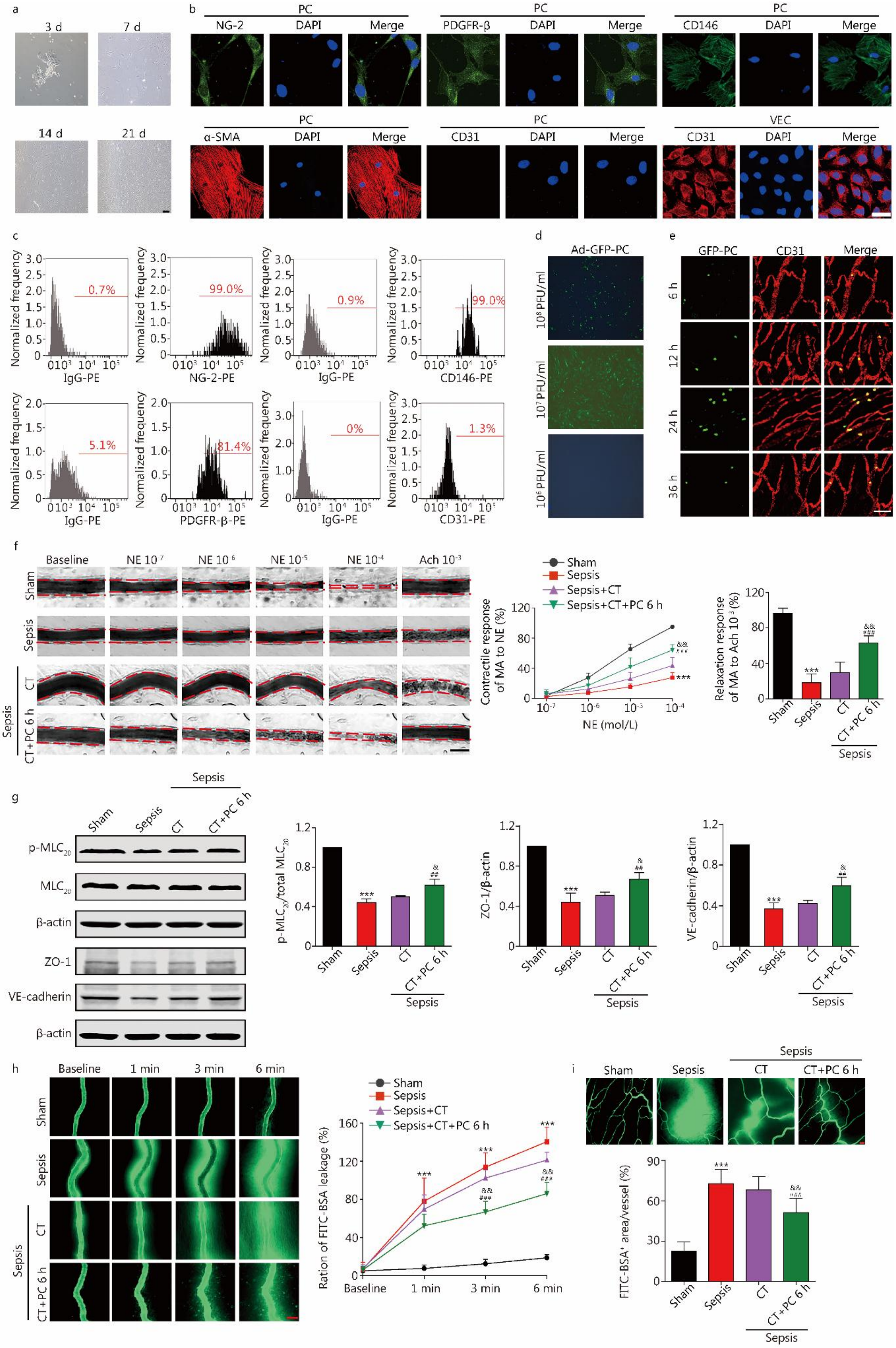

a
3 d
7 d
14 d
21 d
b
PC
NG-2
DAPI
Merge
PDGFR-β
CD146
α-SMA
CD31
VEC
c
Normalized frequency
0.7%
99.0%
0.9%
5.1%
81.4%
0%
1.3%
IgG-PE
NG-2-PE
CD146-PE
PDGFR-β-PE
CD31-PE
d
Ad-GFP-PC
10⁸ PFU/ml
10⁷ PFU/ml
10⁶ PFU/ml
e
GFP-PC
6 h
12 h
24 h
36 h
f
Baseline
NE 10⁻⁷
NE 10⁻⁶
NE 10⁻⁵
NE 10⁻⁴
Ach 10⁻³
Sham
Sepsis
CT
CT+PC 6 h
Sepsis+CT
Sepsis+CT+PC 6 h
Contractile response of MA to NE (%)
NE (mol/L)
Relaxation response of MA to Ach 10⁻³ (%)
g
p-MLC20
MLC20
β-actin
ZO-1
VE-cadherin
p-MLC20/total MLC20
ZO-1/β-actin
VE-cadherin/β-actin
h
1 min
3 min
6 min
Ration of FITC-BSA leakage (%)
i
FITC-BSA⁺ area/vessel (%)

Fig. S4 Primary pericyte identification, the number of pericyte colonization at different times, and the effect of pericyte transplantation on vascular reactivity and barrier function in septic rats within 6 h. **a** Phase contrast photomicrographs of primary rat pericytes in culture. Pericytes migrated out of retinal fragments on day 3; pericytes appeared in primary cultures as large isolated cells or loose colonies on day 7. Pro-confluent primary RMPs showed spread organization on day 14 and 21. Scale bars: 100 μm. **b** Immunofluorescence of pericytes by CLSM. Positive immunostaining of NG-2, PDGFR-β, CD146 and α-SMA and negative immunostaining of CD31 expression in pericytes, and positive immunostaining of CD31 in VECs. Scale bars: 100 μm. **c** Characterization of pericytes by flow cytometry. Grey histogram represents isotype control and black histogram shows the fluorescence intensity of pericytes after incubation with NG-2, CD146, PDGFR-β and CD31 antibodies, respectively. **d** Pericytes were infected with control adenovirus expressing green fluorescent protein (GFP-adenovirus) at different multiplicity of infection. **e** Immunofluorescence by CLSM were used to monitor the GFP-PC location on mesenteric microvascular networks at 6, 12, 24 and 36 h. Scale bars: 50 μm. **f** Changes in vascular response of mesenteric arterioles (n = 8 rats). Scale bars: 50 μm. **g** Expression of p-MLC_{20} in SMA and ZO-1 and VE-cadherin in SMV (n = 3 rats). **h**-**i** Vascular leakage of mesenteric venules and microvascular networks measured at 6 h after pericyte transplantation (n = 8 rats). Scale bars: 50 μm. PC pericyte, NG-2 nerve/glial antigen 2, PDGFR-β platelet-derived growth factor receptor beta, α-SMA α-smooth muscle actin, CLSM confocal laser scanning microscopy, CT conventional treatment, NE norepinephrine, Ach acetylcholine, p-MLC_{20} phosphorylation of myosin light chain 20, ZO-1 zonula occludens-1, VE-cadherin vascular endothelial cadherin, SMV superior mesenteric vein. Data shown as mean ± SD. $^{***}P < 0.001$ vs. Sham; $^{\#\#}P < 0.01$, $^{\#\#\#}P < 0.001$ vs. Sepsis; $^{\&}P < 0.05$, $^{\&\&}P < 0.01$ vs. Sepsis + CT (one-way ANOVA)

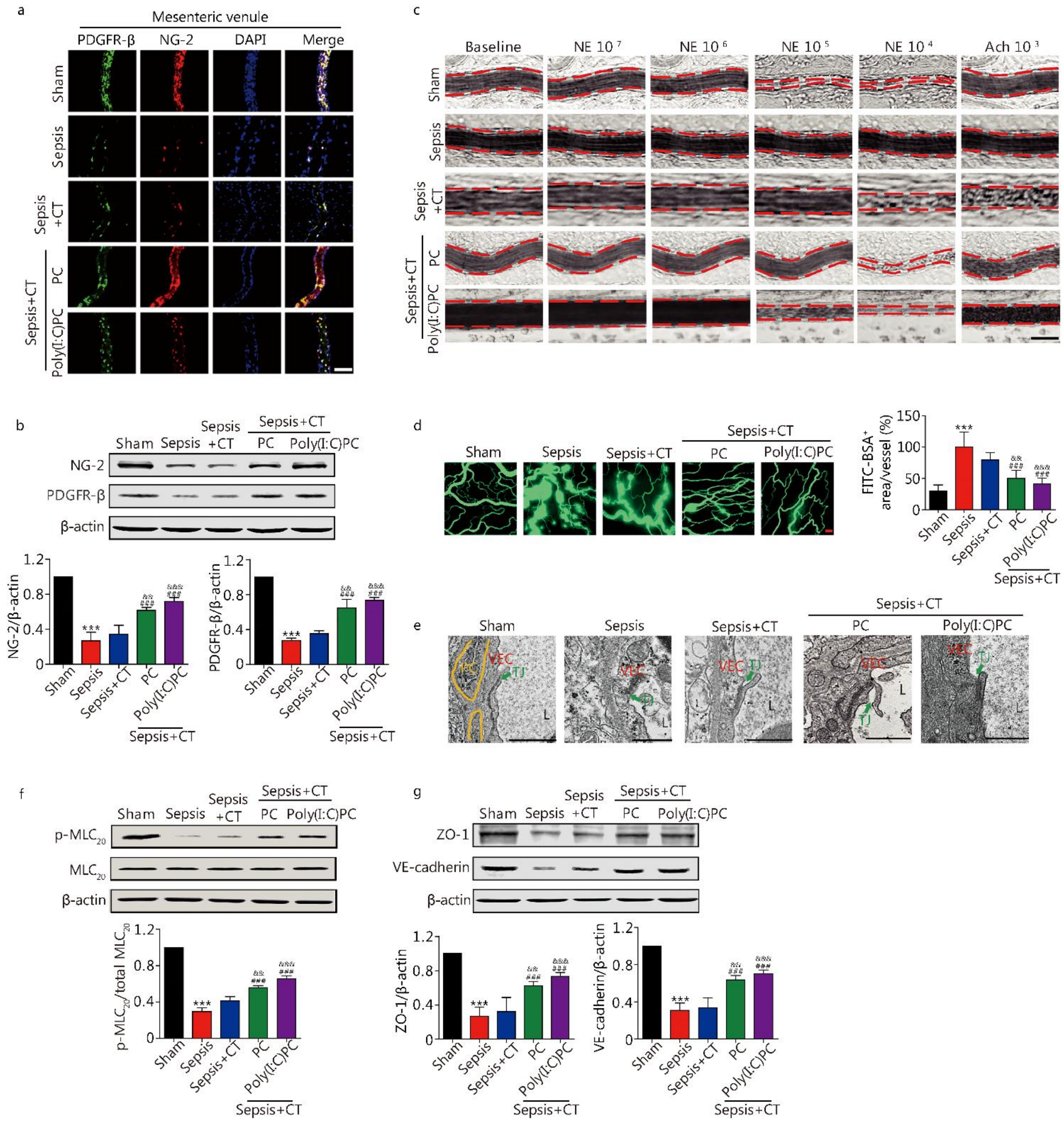

Fig. S5 Pericyte transplantation increases the pericyte coverage and improves the vascular functions at 24 h after sepsis. **a** Mesenteric venules were stained with antibodies against pericytes markers: PDGFR-β (green) and NG-2 (red). Scale bars: 50 μm. **b** Western blotting analysis of NG-2 and PDGFR-β from mesenteric microvascular networks (n = 3). **c** The vascular response of mesenteric arterioles to NE and Ach in vivo after sepsis in rats (n = 8). Scale bars: 50 μm. **d** Vascular leakage of mesenteric microvasular networks measured by the appearance of intravenously injected of FITC–BSA and quantitation of FITC–BSA$^+$ vessel (n = 8 rats). Scale bars: 50 μm. **e** Representative TEM

images of tight junctions (green arrows) of rat retina. Scale bars: 1 μm. **f** Expression of p-MLC_{20} in SMA (n = 3 rats). **g** Expression of ZO-1 and VE-cadherin in SMV (n = 3 rats). PC pericyte, NG-2 nerve/glial antigen 2, PDGFR-β platelet-derived growth factor receptor beta, α-SMA α-smooth muscle actin, CT conventional treatment, NE norepinephrine, Ach acetylcholine, p-MLC_{20} phosphorylation of myosin light chain 20, ZO-1 zonula occludens-1, VE-cadherin vascular endothelial cadherin, SMV superior mesenteric vein, TEM transmission electron microscopy, VEC vascular endothelial cell, TJ tight junction, L lumen. Data shown as mean ± SD. $^{***}P < 0.001$ vs. Sham; $^{\#\#\#}P < 0.001$ vs. Sepsis; $^{\&\&}P < 0.01$, $^{\&\&\&}P < 0.001$ vs. Sepsis + CT (one-way ANOVA)

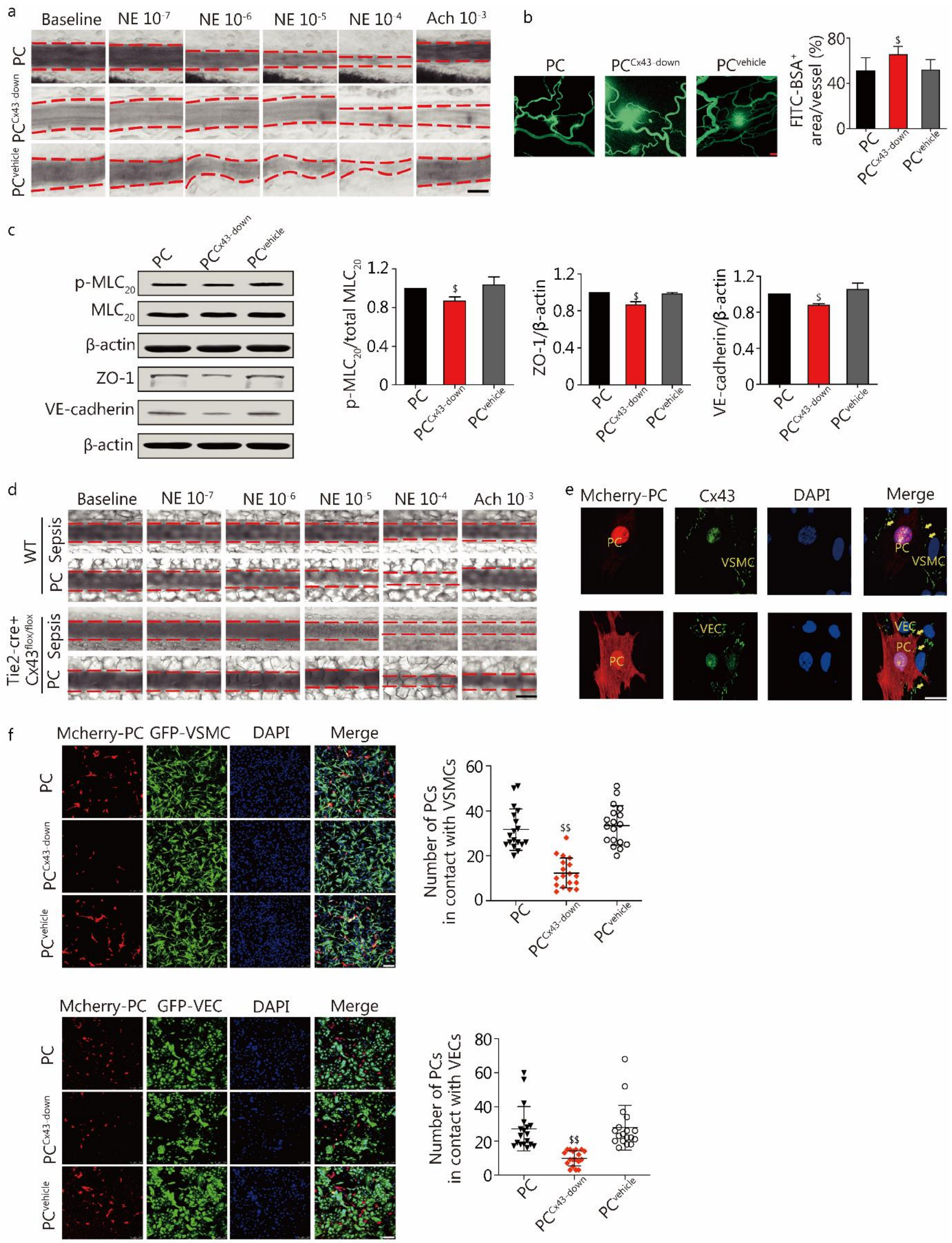

Fig. S6 Effect of Cx43 on vascular reactivity and permeability of VSMCs/VECs through Cx43. **a** Vascular response of mesenteric arterioles to NE and Ach in vivo after PC$^{Cx43\text{-}down}$ transplantation in septic rats (n = 8). Scale bars: 50 μm. **b** Vascular leakage of mesenteric microvascular networks measured after PC$^{Cx43\text{-}down}$ transplantation in septic rats (n = 8). Scale bars: 50 μm. **c** Expression of p-

MLC_{20} in SMA and ZO-1 and VE-cadherin in SMV after $PC^{Cx43\text{-}down}$ transplantation in septic rats (n = 3). **d** Vascular response of mesenteric arterioles to NE and Ach in vivo after sepsis in Tie2-Cre + $Cx43^{flox/flox}$ mice (n = 8). Scale bars: 50 μm. **e** Immunodetection of Cx43 between pericytes (red) and VSMCs or VECs (green). Scale bars: 20 μm. Cx43 protein is immunolabeled (yellow arrows). **f** Number of pericytes attached to the VSMCs/VECs. Scale bars: 100 μm. VSMCs vascular smooth muscle cells, VECs vascular endothelial cells, PC pericyte, NE norepinephrine, Ach acetylcholine, p-MLC_{20} phosphorylation of myosin light chain 20, SMA superior mesenteric artery, ZO-1 zonula occludens-1, VE-cadherin vascular endothelial cadherin, SMV superior mesenteric vein. Data shown as mean ± SD. $^{\$}P < 0.05$, $^{\$\$}P < 0.01$ vs. PC (one-way ANOVA)

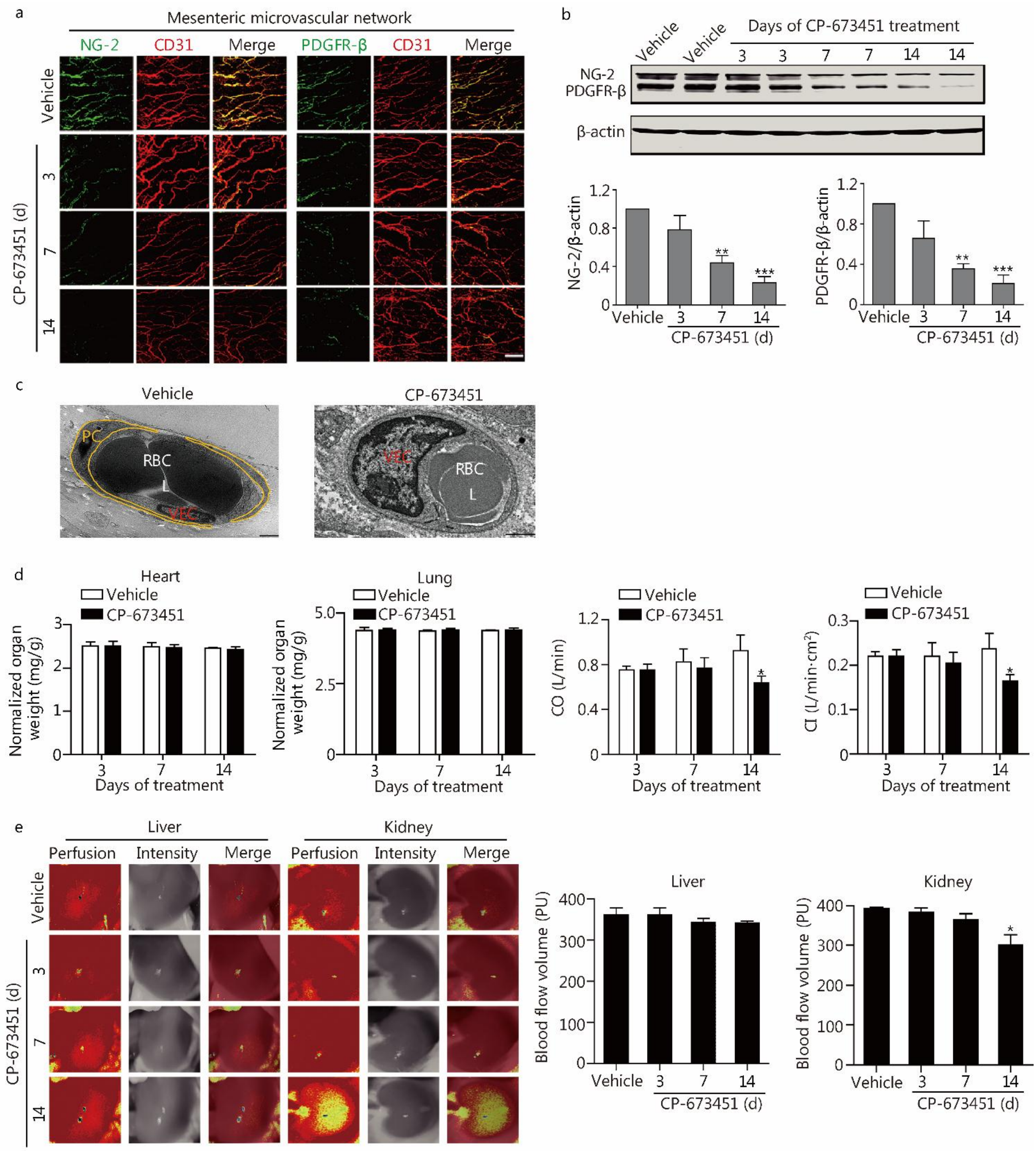

Fig. S7 Depletion of pericytes with CP-673451. **a** Confocal micrographs from representative mesenteric sections showing staining for NG-2 (pericyte marker; green), PDGFR-β (pericyte marker; green) and CD31 (VEC marker; red) from rats treated with CP-673451 [40 mg/(kg·d)] or PEG-400 vehicle for 3, 7 or 14 d. Scale bars: 100 μm. **b** Western blotting analysis of NG-2 and PDGFR-β from mesenteric microvessel of rats treated with CP-673451 or vehicle for 3, 7 or 14 d (n = 3). **c** Representative TEM images of pericytes in mesenteric microvessel. Scale bars: 1 μm. **d** Heart weight

or lung weight to body weight ratios, CO and CI (*n* = 5 rats). **e** The blood flows of liver and kidney were measured (*n* = 5 rats). PC pericyte, CO cardiac output, CI cardiac index, NG-2 nerve/glial antigen 2, PDGFR-β platelet-derived growth factor receptor beta, TEM transmission electron microscopy, VEC vascular endothelial cell, RBC red blood cell, L lumen. Data shown as mean ± SD. $^{*}P < 0.05$, $^{**}P < 0.01$, $^{***}P < 0.001$ vs. Vehicle (one-way ANOVA)

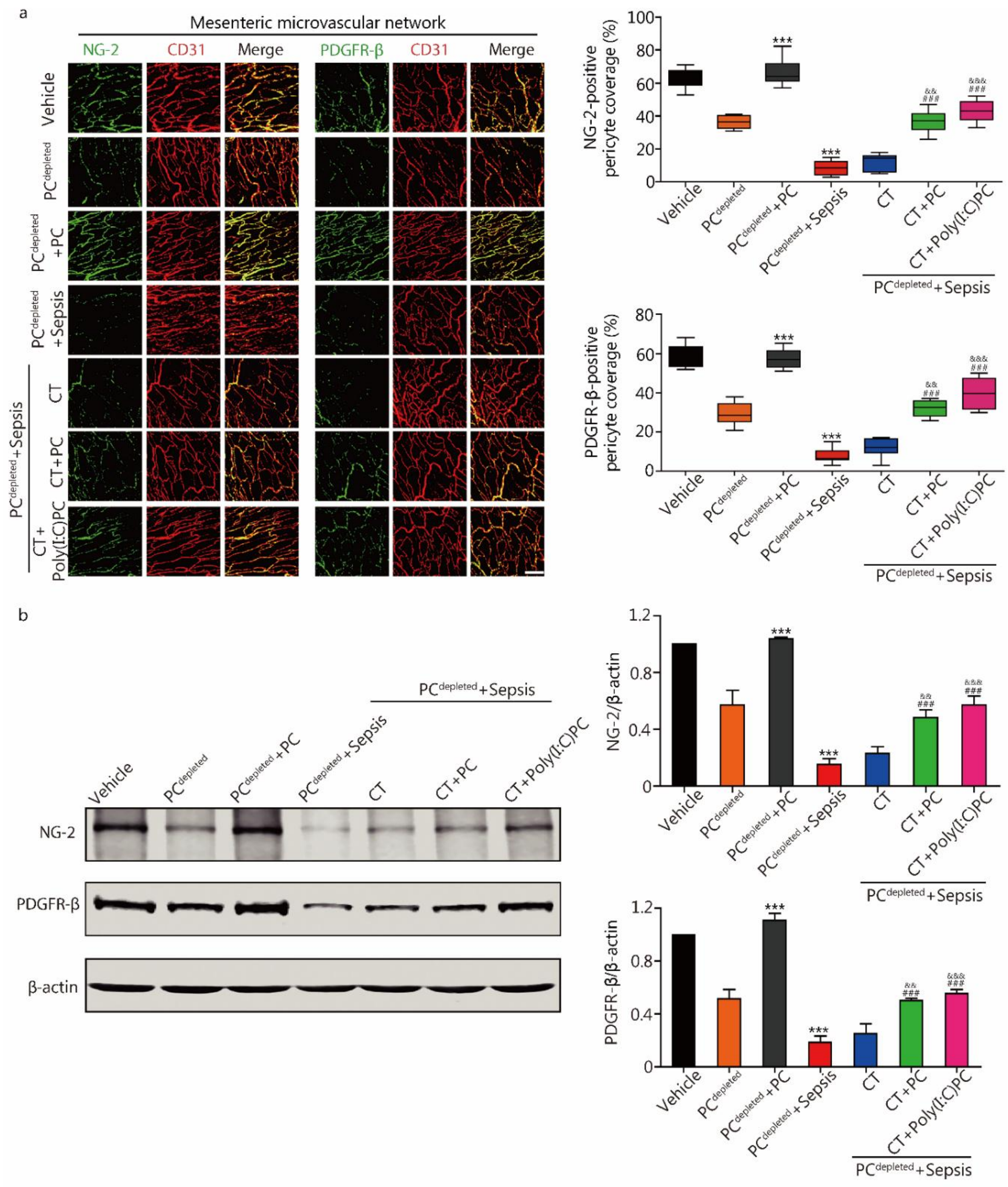

Fig. S8 Pericyte depletion aggravates sepsis-induced pericyte loss, which in turn is rescued by pericyte transplantation. **a** Pericytes in the mesenteric microvascular networks were stained for NG-2, PDGFR-β, and CD31 (n = 8 rats). Scale bars: 100 μm. **b** Western blotting analysis of NG-2 and PDGFR-β from mesenteric microvascular networks (n = 3 rats). PC^{depleted} group: pericyte-depleted rats. NG-2 nerve/glial antigen 2, PDGFR-β platelet-derived growth factor receptor beta, PC pericyte, CT conventional treatment. Data shown as mean ± SD. $^{***}P < 0.001$ vs. PC^{depleted}; $^{\#\#\#}P < 0.001$ vs. PC^{depleted} + Sepsis; $^{\&\&}P < 0.01$, $^{\&\&\&}P < 0.001$ vs. PC^{depleted} + Sepsis + CT (one-way ANOVA)

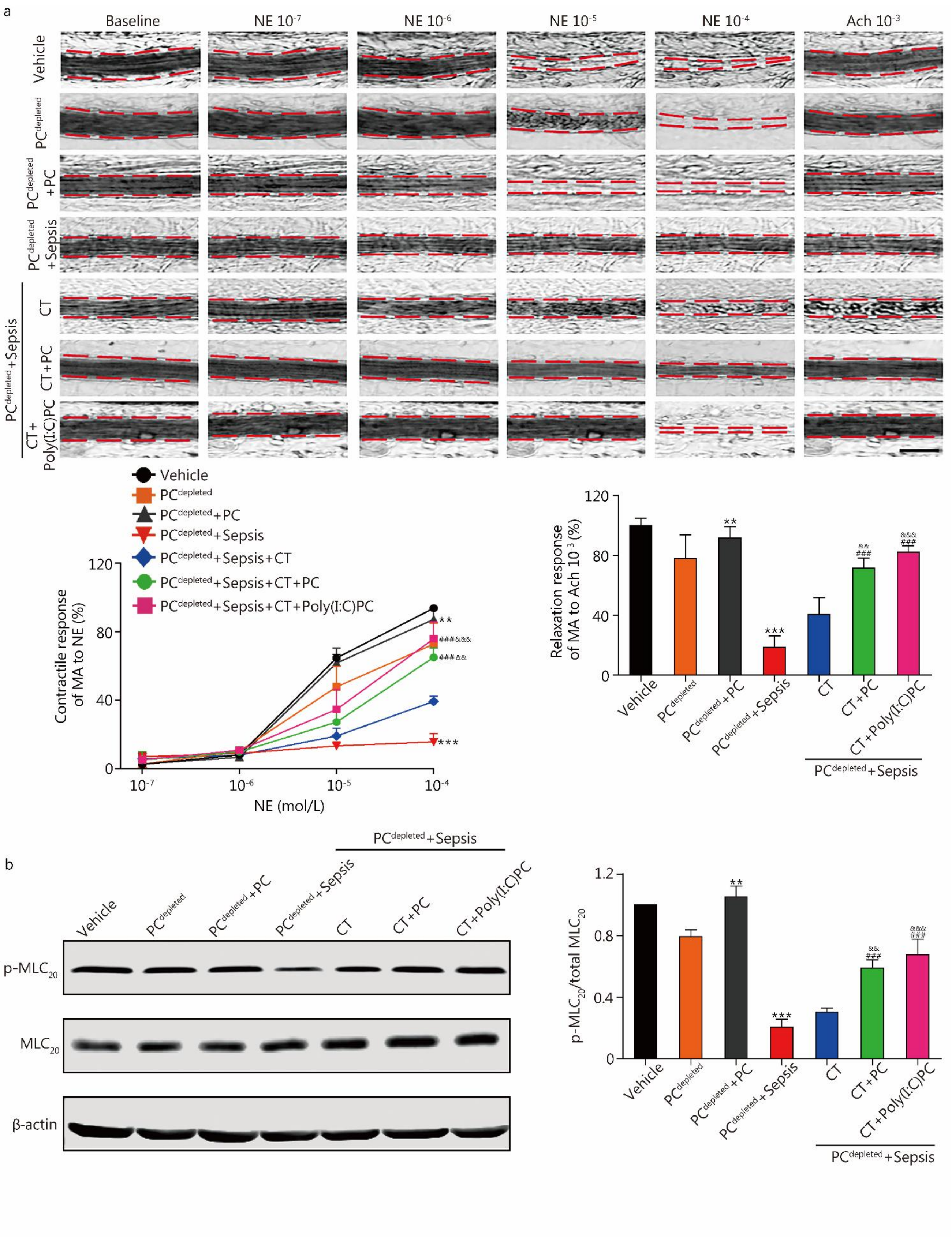

Fig. S9 Pericyte depletion aggravates sepsis-induced vascular hyporeactivity, which in turn is rescued by pericyte transplantation. **a** Changes in vascular response of mesenteric arterioles and vascular

leakage of mesenteric venules in pericyte-depleted rats after pericyte transplantation (n = 8 rats). Scale bars: 50 μm. **b** Expression of p-MLC_{20} in SMA (n = 3 rats). PC pericyte, CT conventional treatment, SMA superior mesenteric artery, NE norepinephrine, Ach acetylcholine, MA mesenteric arteriole. Data shown as mean ± SD. $^{**}P < 0.01$, $^{***}P < 0.001$ vs. $PC^{depleted}$; $^{\#\#\#}P < 0.001$ vs. $PC^{depleted}$ + Sepsis; $^{\&\&}P < 0.01$, $^{\&\&\&}P < 0.001$ vs. $PC^{depleted}$ + Sepsis + CT (one-way ANOVA)

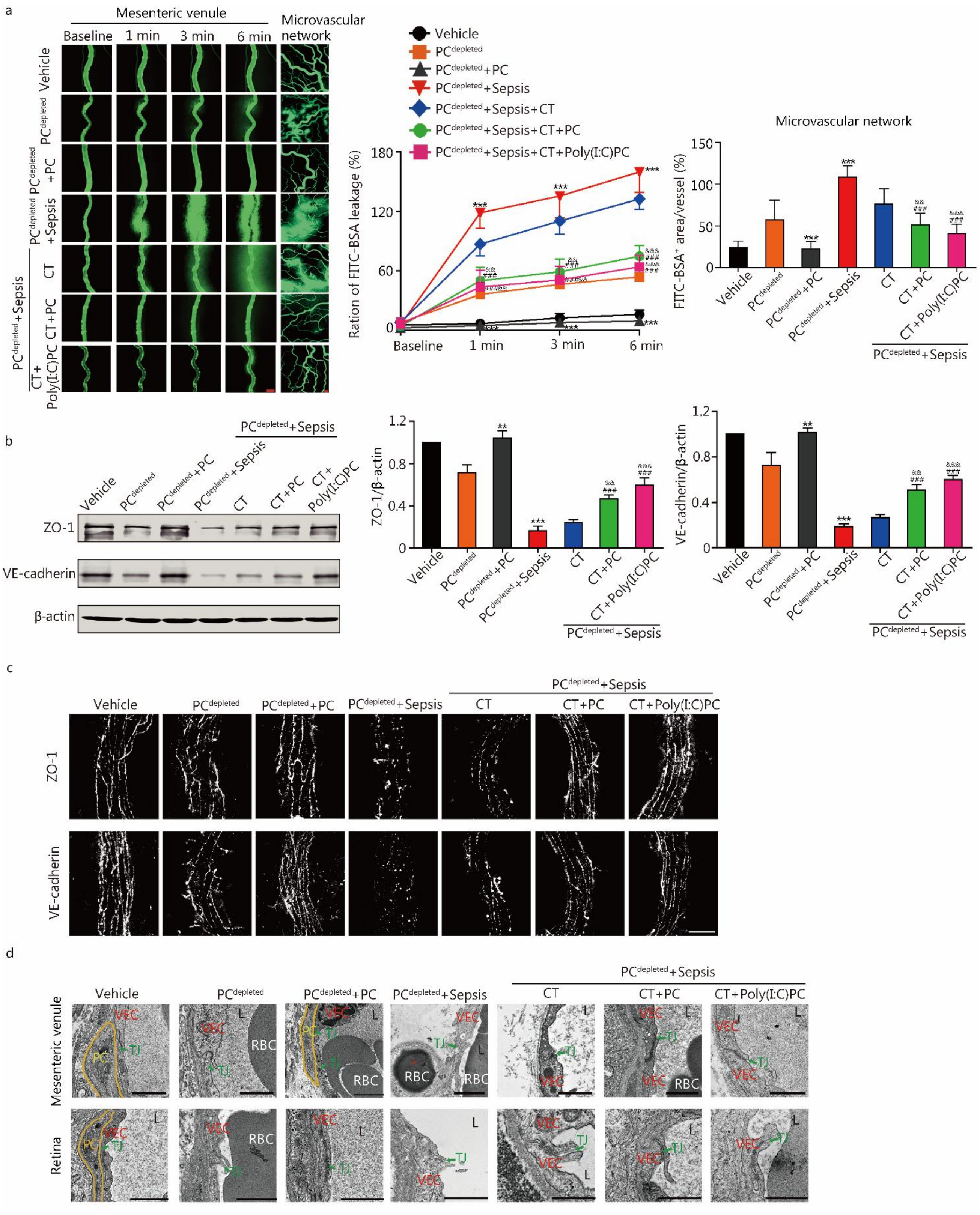

Fig. S10 Pericyte depletion aggravates sepsis-induced vascular leakage, which in turn is rescued by pericyte transplantation. **a** Vascular leakage of mesenteric venules and microvascular networks (*n* = 8 rats). Scale bars: 50 μm. **b** Expression of ZO-1 and VE-cadherin in SMV (*n* = 3 rats). **c** Immunohistochemistry for ZO-1 and VE-cadherin in rat mesenteric venule. Scale bars: 20 μm. **d**

Representative TEM images of tight junctions in mesenteric venule and retina (green arrows indicate the tight junctions, * indicate the erythrocyte diapedesis). Scale bars: 1 µm. VEC vascular endothelial cell, RBC red blood cell, TJ tight junction, L lumen, PC pericyte, CT conventional treatment, ZO-1 zonula occludens-1, VE-cadherin vascular endothelial cadherin, TEM transmission electron microscopy, SMV superior mesenteric vein. Data shown as mean ± SD. $^{**}P < 0.01$, $^{***}P < 0.001$ vs. $PC^{depleted}$; $^{\#\#\#}P < 0.001$ vs. $PC^{depleted}$ + Sepsis; $^{\&\&}P < 0.01$, $^{\&\&\&}P < 0.001$ vs. $PC^{depleted}$ + Sepsis + CT (one-way ANOVA)

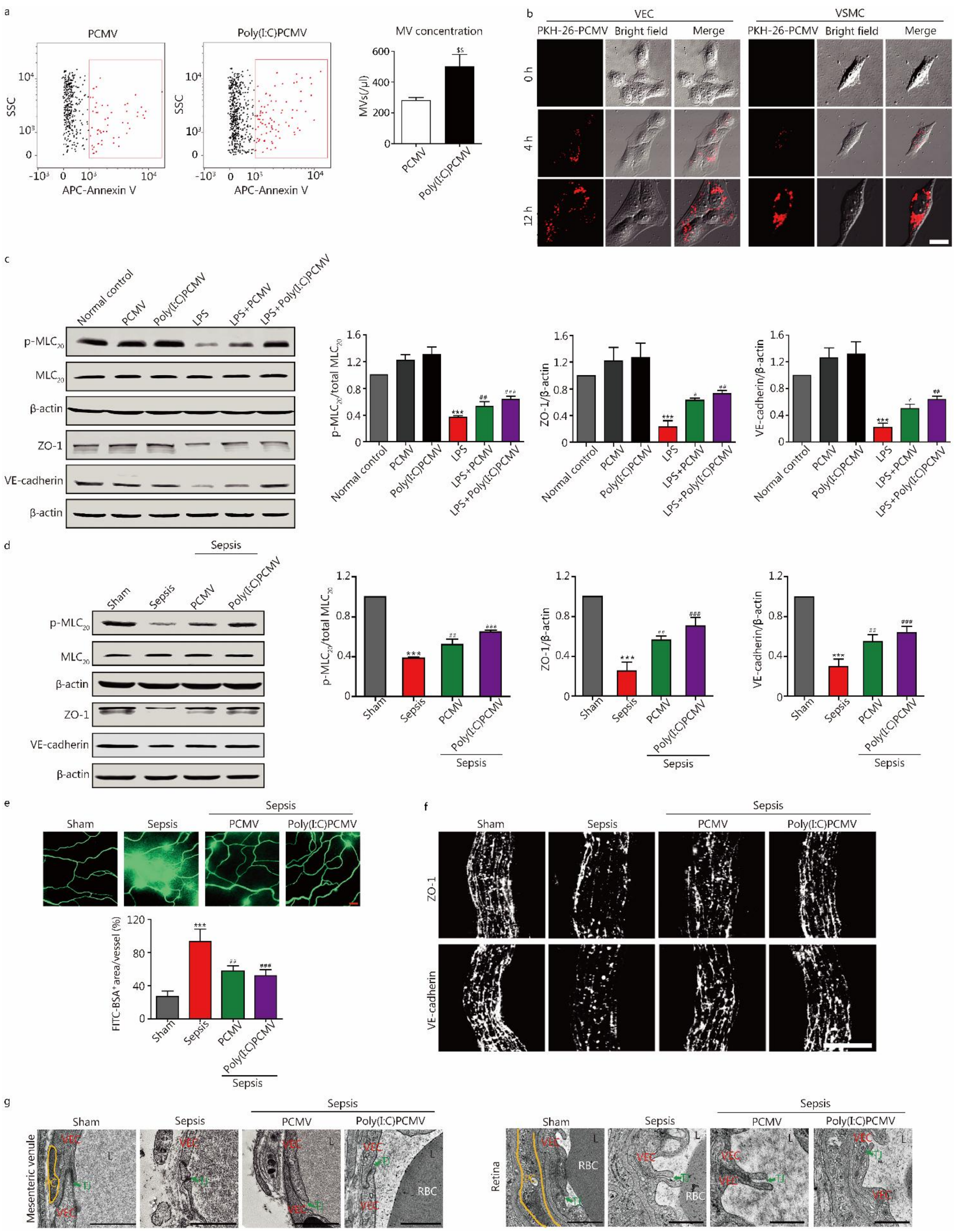

Fig. S11 PCMVs improves the contractile response of VSMCs and barrier function of VECs after sepsis. **a** Flow cytometric detection of PCMVs and Poly(I:C)PCMVs. **b** PCMVs labeled with PKH-26

were added into VECs and VSMCs, and internalization of microvesicles were measured at different time. **c** VSMCs and VECs treated with PCMVs were analyzed by Western blotting analysis (*n* = 3 cells). Scale bars: 20 μm. **d** Expression of p-MLC_{20} in SMA and ZO-1 and VE-cadherin in SMV (*n* = 3 rats). **e** Vascular leakage of mesenteric microvascular networks (*n* = 8 rats). Scale bars: 50 μm. **f** Immunohistochemistry for ZO-1 and VE-cadherin in mesenteric venules. Scale bars: 20 μm. **g** Representative TEM images of tight junctions in mesenteric venules and retina (green arrows indicate the tight junctions). Scale bars: 1 μm. PC pericyte, PCMV pericyte-derived microvesicle, SSC side scatter, APC allophycocyanin, MVs microvesicles, VEC vascular endothelial cell, VSMC vascular smooth muscle cell, LPS lipopolysaccharides, p-MLC_{20} phosphorylation of myosin light chain 20, ZO-1 zonula occludens-1, VE-cadherin vascular endothelial cadherin, SMA superior mesenteric artery, SMV superior mesenteric vein, RBC red blood cell, TJ tight junction, L lumen, TEM transmission electron microscopy. Data shown as mean ± SD. $^{\$\$}P < 0.01$ vs. PCMV; $^{***}P < 0.001$ vs. Sham; $^{\#}P < 0.05$, $^{\#\#}P < 0.01$, $^{\#\#\#}P < 0.001$ vs. Sepsis or LPS (one-way ANOVA)

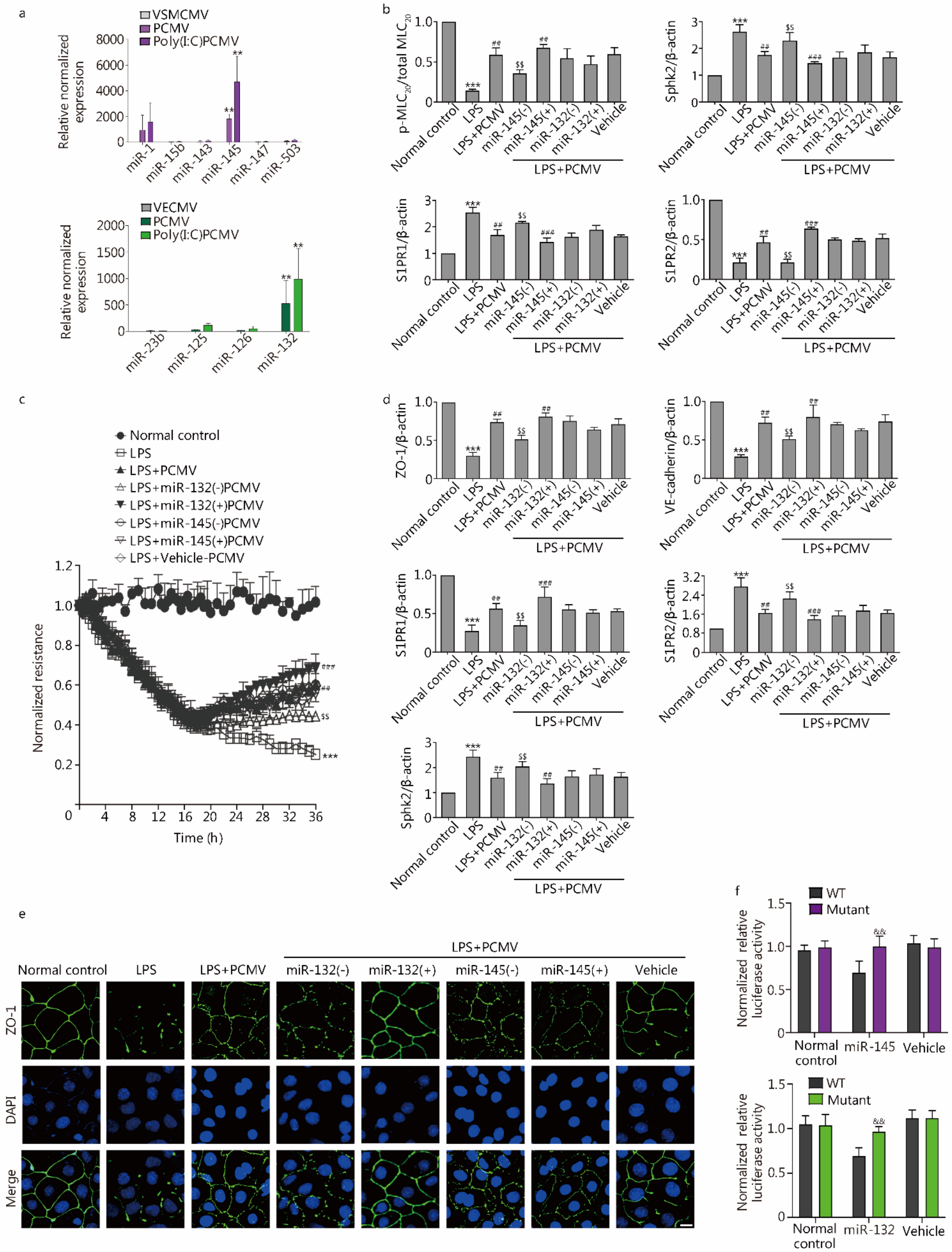

Fig. S12 PCMVs carry miR-145 and miR-132 to VSMCs and VECs to orchestrate effects on the contractile response of VSMCs and barrier function of VECs. **a** Comparison of microRNAs expression

in PCMVs isolated from pericytes treated with or without Poly(I:C) ($n = 3$). **b** Quantitative analysis of protein expression in **Fig. 5b**. **c** VECs treated with different types of PCMVs were added into VECs, and TEER of each group was measured ($n = 3$ cells). **d** Quantitative analysis of protein expression in **Fig. 5d**. **e** VECs treated with different types of PCMVs were analyzed by immunofluorescence for ZO-1. Scale bars: 20 μm. **f** Effects of miR-145 and miR-132 on the Sphk2 and mutant Sphk2 in VSMCs and VECs, respectively, measured by dual-luciferase system. PC pericyte, PCMV pericyte-derived microvesicle, VEC vascular endothelial cell, VSMC vascular smooth muscle cell, VSMCMV VSMC-derived microvesicle, VECMV VEC-derived microvesicle, p-MLC_{20} phosphorylation of myosin light chain 20, Sphk2 sphingosine kinase 2, S1PR1 sphingosine-1-phosphate receptor 1, LPS lipopolysaccharides, TEER transendothelial electrical resistance, ZO-1 zonula occludens-1, VE-cadherin vascular endothelial cadherin, WT wide type. Data shown as mean ± SD. $^{**}P < 0.01$, $^{***}P < 0.001$ vs. VSMCMV or VECMV or Normal control; $^{\#\#}P < 0.01$, $^{\#\#\#}P < 0.001$ vs. LPS; $^{\$\$}P < 0.01$ vs. LPS + PCMV; $^{\&\&}P < 0.01$ vs. WT (one-way ANOVA)
